# Identification and structure-based drug design of cell-active inhibitors of interleukin 17A at a novel C-terminal site

**DOI:** 10.1038/s41598-022-18760-1

**Published:** 2022-08-26

**Authors:** Eric R. Goedken, Maria A. Argiriadi, Justin D. Dietrich, Andrew M. Petros, Navasona Krishnan, Sanjay C. Panchal, Wei Qiu, Haihong Wu, Haizhong Zhu, Ashley M. Adams, Pierre M. Bodelle, Lucas Goguen, Paul L. Richardson, Peter F. Slivka, Myron Srikumaran, Anup K. Upadhyay, Bainan Wu, Russell A. Judge, Anil Vasudevan, Sujatha M. Gopalakrishnan, Philip B. Cox, Vincent S. Stoll, Chaohong Sun

**Affiliations:** 1 AbbVie Bioresearch Center, 100 Research Drive, Worcester, MA 01605 USA; 2 AbbVie Inc., 1 North Waukegan Rd., North Chicago, IL 60064 USA; 3 Former AbbVie Employee, North Chicago, USA

**Keywords:** Drug screening, Medicinal chemistry, Cytokines, Drug discovery, Inflammation, NMR spectroscopy, X-ray crystallography

## Abstract

Anti-IL17A therapies have proven effective for numerous inflammatory diseases including psoriasis, axial spondylitis and psoriatic arthritis. Modulating and/or antagonizing protein–protein interactions of IL17A cytokine binding to its cell surface receptors with oral therapies offers the promise to bring forward biologics-like efficacy in a pill to patients. We used an NMR-based fragment screen of recombinant IL17A to uncover starting points for small molecule IL17A antagonist discovery. By examining chemical shift perturbations in 2D [^1^H, ^13^C-HSQC] spectra of isotopically labeled IL17A, we discovered fragments binding the cytokine at a previously undescribed site near the IL17A C-terminal region, albeit with weak affinity (> 250 µM). Importantly this binding location was distinct from previously known chemical matter modulating cytokine responses. Subsequently through analog screening, we identified related compounds that bound symmetrically in this novel site with two copies. From this observation we employed a linking strategy via structure-based drug design and obtained compounds with increased binding affinity (< 50 nM) and showed functional inhibition of IL17A-induced cellular signaling (IC_50_~1 µM). We also describe a fluorescence-based probe molecule suitable to discern/screen for additional molecules binding in this C-terminal site.

## Introduction

The Interleukin 17 (IL17) family comprises several structurally-related dimeric cytokines that are elicited by infection and other immunological stimuli^1^. IL17 proteins drive and amplify inflammatory responses in a wide variety of cells including keratinocytes, fibroblasts and lymphocytes^2^. The preeminent family member is IL17A^3^, which forms homodimers and can also heterodimerize with IL17F. The IL17A homodimer can amplify the action of other inflammatory cytokines such as TNF or IL-1β. By binding to a functional cell surface receptor composed of IL17 receptor A (IL17RA) and receptor C (IL17RC) chains, which are broadly expressed, IL17A can stimulate cellular signaling via the Act1 protein^4–7^. The resulting IL17-directed events ultimately lead to the coordinated release of cytokines and chemokines such as IL-6, IL-8, and CXCL1 but also defensin proteins and anti-microbial peptides that collectively contribute important cues to maintaining and amplifying defensive response to pathogens.

When dysregulated, however, the inappropriate activation of IL17 can also contribute to numerous inflammatory and autoimmune disorders^8–10^. Indeed, antibodies neutralizing IL17A have been shown to be powerful therapeutics for human diseases and positive clinical trial data has been reported in psoriasis, psoriatic arthritis, ankylosing spondylitis with now approved drugs such as secukinumab/Cosentyx and ixekizumab/Taltz^11–13^. An antibody targeting the cell surface portion of the IL17 receptor A chain (Brodalumab/Siliq) has also been approved for use. Given these observations, there is great interest in finding additional drugs in this pathway and indeed numerous other anti-IL17 biologics are currently under investigation^14^. While such molecules have shown good efficacy in treating psoriasis, one disadvantage is that these antibodies require administration by injection, which many patients dislike affecting overall compliance. Another disadvantage is that antibodies typically exhibit long serum half-lives (often weeks) and therefore, drug action cannot be quickly removed/attenuated in situations (like infection) requiring the restoration of functional IL17 to resolve. As such, small molecule antagonists disrupting IL17A function that are suitable for daily, oral dosing represent an attractive option for the next generation of IL17-based therapies.

**Figure 1:**
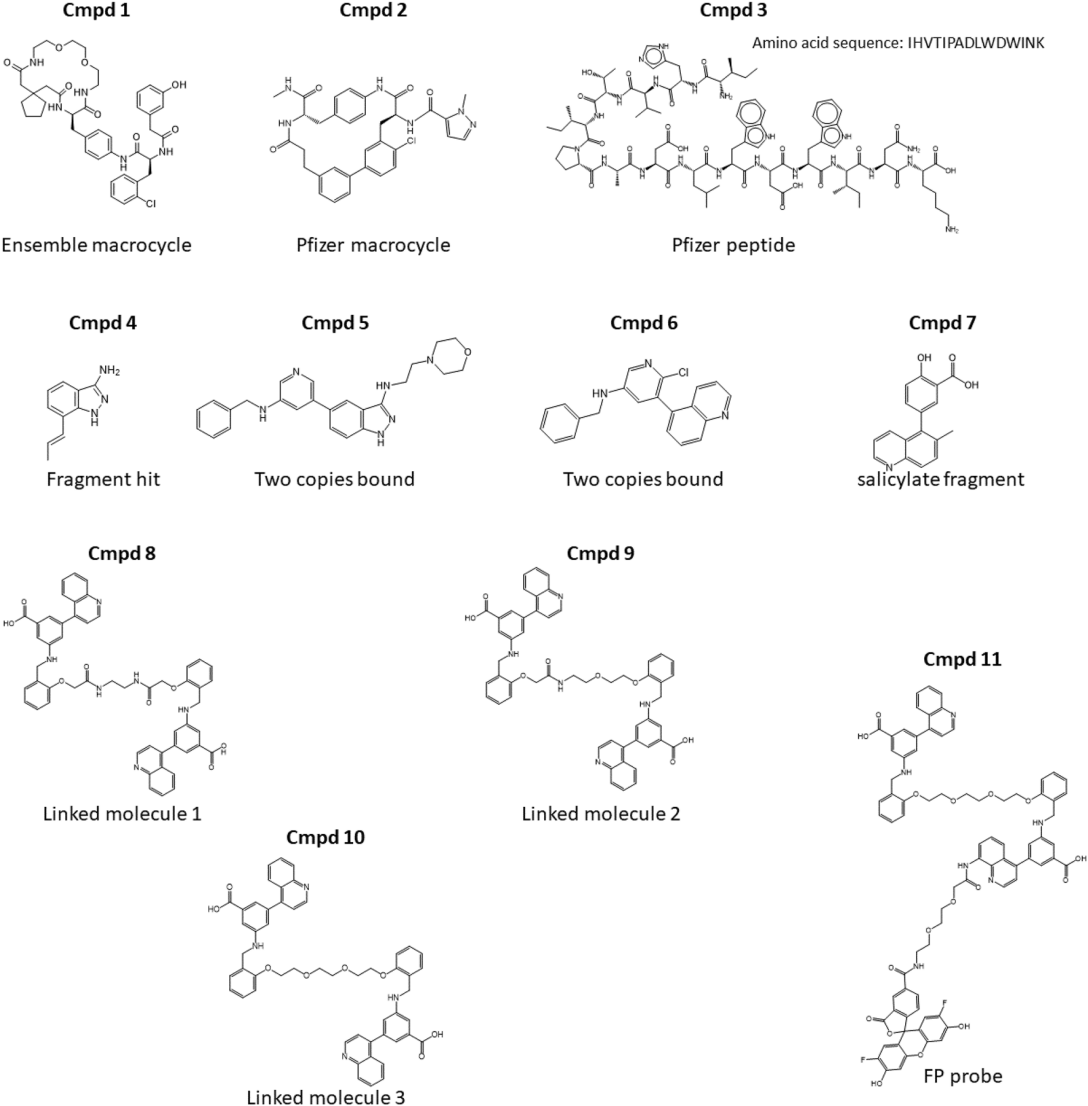
Compound structures.

**Figure 2:**
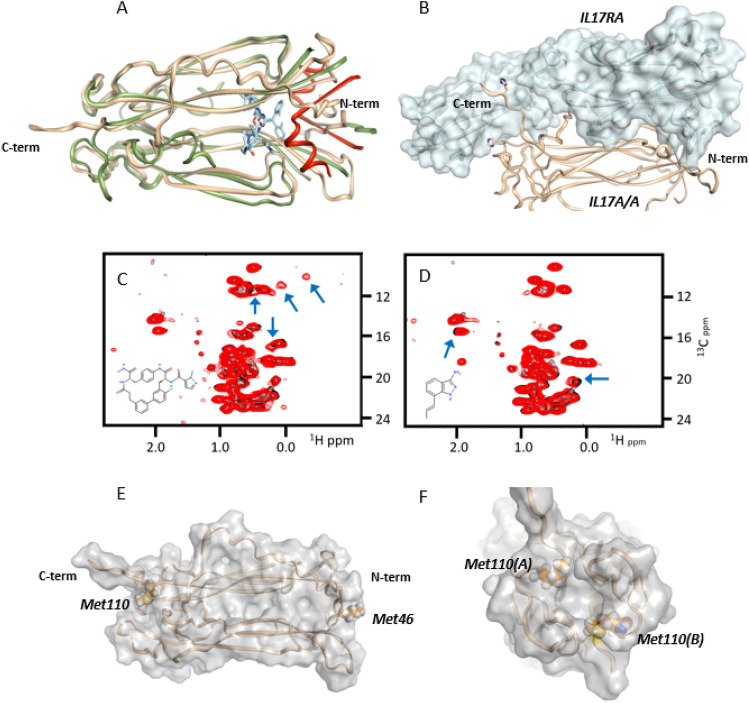
IL17A binding sites and discovery of novel binding site by NMR based fragment screening. (**A**) Overlay of apo IL17A^21^ (tan ribbon) with complexed IL17A structure^16^ (green ribbon; PDB 5HI3) with Pfizer macrocycle (Cmpd 2; light blue: Pfizer macrocycle) and Pfizer linear peptide^18^ (Cmpd 3; red). (**B**) IL17A complexed with IL17 Receptor A^21^ (PDB 4HSA). (**C**,**D**) ^13^C-HSQC spectra (recorded at 600 MHz) of IL17A isotopically labeled at the methyl groups of isoleucine (δ), valine, leucine and methionine. (**C**) Overlay of spectra recorded in the absence (black) and presence (red) of Pfizer macrocycle (Cmpd 2). (**D**) Overlay of spectra recorded in the absence (black) and presence (red) of Cmpd 4. In each case, blue arrows highlight observed perturbations upon compound binding. (**E**) Ribbon structure of IL-7A dimer showing Met46 and Met110 as sticks or spheres. (**F**) “End view” of IL17A C-terminus showing potential pocket for fragment binding.

While obtaining high quality protein–protein interaction inhibitors (PPI) with small molecules remains a great challenge, several preclinical small molecule IL17A inhibitors have been previously described. These include macrocyclic molecules from Ensemble Therapeutics^15^ and Pfizer^16^ (Cmpds 1 and 2; Fig. 1). The Pfizer macrocycle has been shown via x-ray crystallographic studies to bind in a deep pocket at the dimer interface of IL17A^16^ (Fig. 2A). This pocket is a key site for interaction of the IL17A dimer with its cell surface receptors^17^ (Fig. 2B). Furthermore, these macrocycles, as well as linear peptide-based inhibitors, which bind at an adjacent N-terminal site^18^ (Fig. 2A), have been shown to block IL17A signaling in cells. However, these compounds have not yet advanced into human clinical trials.

Here we present a new class of inhibitor molecules that interact with IL17A at a novel C-terminal binding site. These inhibitors were discovered and characterized through NMR-based and Surface Plasmon Resonance (SPR)-based fragment screening. Coupled with a variety of biochemical and cellular experiments to measure their impact on IL17 function and relying on structure-based drug design to improve their affinity, we were able to further optimize early leads to obtain cell-active molecules specifically disrupting IL17 action by occluding an entirely separate (and novel) location on the IL17A molecule. As such, these early molecules point the way toward a new route to IL17 small molecule antagonists.

## Results

When we initially undertook our studies aimed at discovering a small-molecule inhibitor of IL17A signaling, the only known, small-molecule, inhibitor was that from Ensemble Therapeutics^15^ (Cmpd 1; Fig. 1). While this macrocycle proved to be a potent IL17A binder, there was no available structural information as to where exactly this molecule bound to the IL17A dimer. Subsequent to this work, researchers at Pfizer published the structure of both their macrocyclic IL17A inhibitor (Cmpd 2, Fig. 2A), which penetrates deeply into a pocket formed at the IL17A dimer interface, disrupts the dimer symmetry^16^, and directly overlaps with the IL17 receptor binding site (Fig. 2B) and their linear peptide-based inhibitor (Cmpd 3), which binds at an adjacent site near the N-terminus^18^, Fig. 2A.

### IL17A NMR-based fragment screen indicates two binding regions

The Ensemble and Pfizer macrocycles bind potently to the IL17A dimer with *K*_D_s for both ~ 4 nM as determined by Surface Plasmon Resonance (SPR). However, due to their large size, (molecular weights 776 and 613 Da respectively), they have low binding efficiency indices (BEI) ~ 11–13^19^. Despite low nM affinity, we and others have found that this class of IL17 inhibitors often show weaker potency in cellular assays (typically 0.3 to 5 µM)^16^.

In order to discover potent, novel IL17A binders we undertook an NMR-based fragment screen^20^. To enable the screen, we isotopically labeled IL17A protein via expression in *E. coli* with ^13^C at the methyl groups of isoleucine (δ only), leucine, valine, and methionine. The fragment screen was carried out by recording two-dimensional [^1^H, ^13^C]-HSQC spectra of the isotopically labeled IL17A protein in the presence of potential fragment binders. A library of ~ 4,000 fragments was screened – initially in mixtures of 12, with each fragment at 1 mM in the NMR tube. Those mixtures that induced significant chemical shift perturbations (CSPs; ≥ 0.02 ppm ^1^H or ≥ 0.15 ppm ^13^C) with respect to the ligand-free reference spectrum were then deconvoluted to determine the individual fragment binders.

This NMR-based screen yielded several robust fragment binders, some of which induced very similar CSPs as we observed upon addition of the Pfizer compound to the protein (Fig. 2C). Another set of binders, the most interesting of which was an aminoindazole (Cmpd 4), induced CSPs quite distinct from those induced by the Pfizer compound (Fig. 2D). Most notably, binding of these fragments perturbed a methionine methyl resonance not perturbed upon Pfizer compound addition, suggesting that the binding site for these fragments was proximal to a methionine sidechain, in a region of the protein distinct from the open groove engaged by the Pfizer macrocycle. The IL17A protein has a methionine residue at position 46, near the N-terminus, and at position 110, near the C-terminus (Fig. 2E). In addition, our screening protein construct has an artifactual methionine residue at the N-terminus as a result of *E. coli* expression.

A close inspection of the unliganded IL17A dimer^21^ revealed that a small hydrophobic pocket with the sidechain of Met110 on one side appears to be capable of accommodating a fragment-sized molecule (Fig. 2F). In contrast, the sidechain of Met46 points directly into solvent away from the core of the dimer. Based on this observation, our hypothesis was that the set of fragments including the aminoindazole that perturb the methionine methyl resonance upon binding to IL17A likely bind into the C-terminal pocket formed in part by Met110. In order to confirm this hypothesis, we prepared ^13^C-labeled protein in which Met46 and Met110 were separately mutated to alanine. An overlay of the ^13^C-HSQC spectrum of the Met110Ala mutant protein with that of the wild type protein confirmed that the perturbed methionine resonance arises from Met110 and thus verified that the aminoindazole fragment binds into this C-terminal pocket (Supplemental Fig. 1).

With the binding location confirmed for the aminoindazole fragment, we then determined the affinity of this fragment for IL17A using surface plasmon resonance (SPR). This fragment bound with relatively weak affinity (*K*_D_ > 250 µM) to IL17A. We subsequently utilized a combination of SPR and 2D NMR to drive fragment optimization of this core (see Supplemental Figs. 2 and 3). Initial rounds of analoging focused on identifying additional indazole-containing fragments and lead-like molecules (MW < 450 Da) from our compound repository. From this exercise, we identified Cmpd 5 that was flanked off the 3-position with a morpholinoethyl amine tail and at the 5-position by a benzylaminopyridine moiety. This compound showed robust binding by 2D NMR and upon titration, displayed an NMR *K*_D_ of 12 µM. Leveraging this information, a second round of analoging against our compound collection focused on expanding diversity around the 5-benzylaminopyridine moiety in Cmpd 5. From this, we identified Cmpd 6, which also showed robust binding by 2D NMR. We found this compound gave an SPR-determined *K*_D_ ~ 70 µM. Both analogs, upon addition to the IL17A dimer, elicited very similar perturbations in the NMR spectrum to the original fragment core, indicating that they both bind in the same C-terminal site. In reviewing changes in the methyl region of 13C-HSQC spectrum of Cmpd 6 bound to IL17A compared that of unliganded IL17A, we found the resonance from Met110 completely broadened out in the presence of Cmpd 6, consistent with the prior change observed in this region from the earlier hit Cmpd 4 (Supplemental Fig. 3).

**Figure 3:**
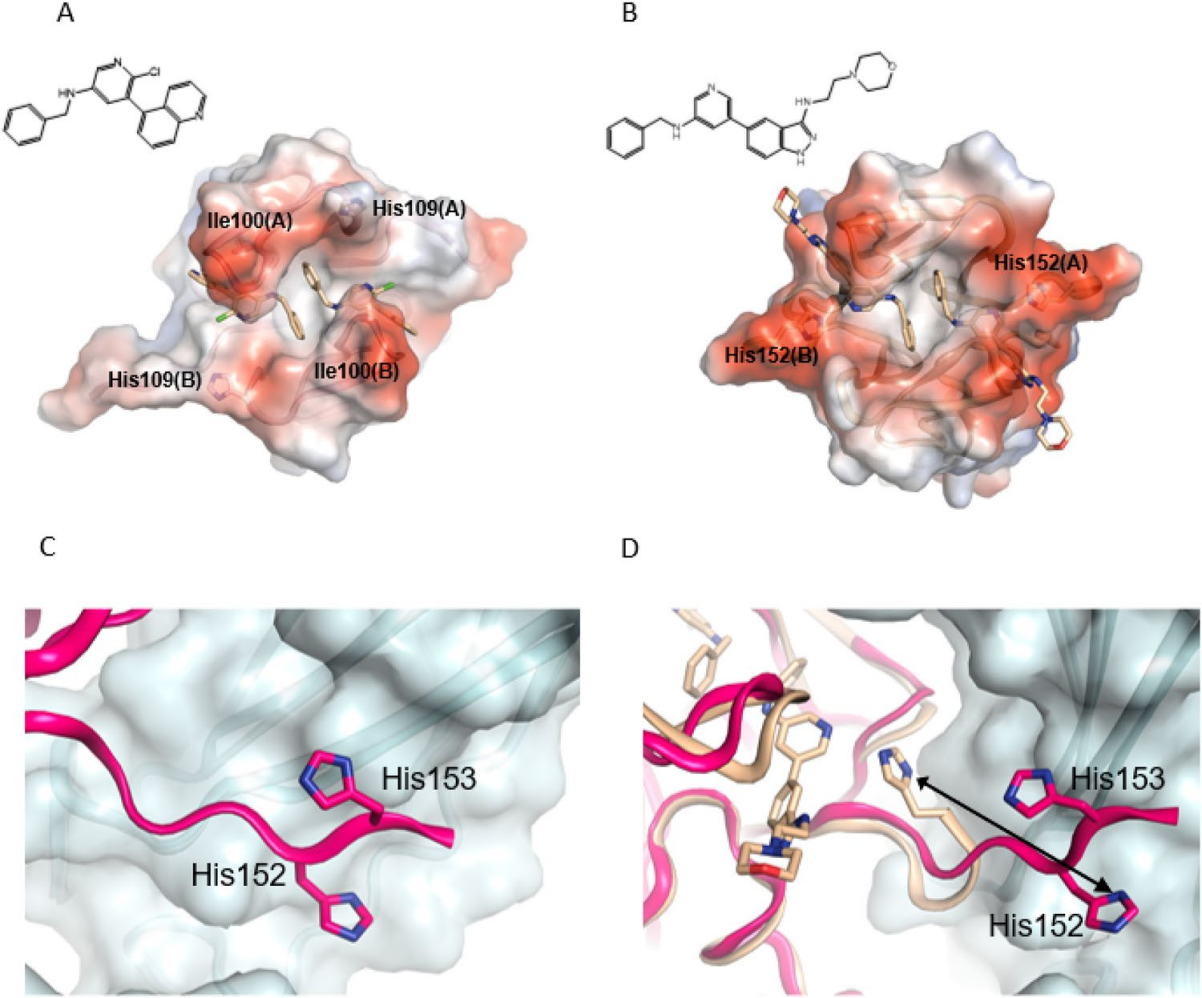
(**A**) Structures of Cmpd 6 and (**B**) Cmpd 5 bound to IL17A C-terminal site and highlighted interactions. (**C,D**) Evidence of C-terminal importance was seen in these crystal structures with respect to positioning of His152 (**C**) “Extended position” of His152 in IL17A complex with Cmpd 6 (**D**) Overlay of Cmpd 5 (light orange structure with movement toward His152). In both (**C**,**D**) the position of the reported IL17 receptor A as bound to IL17A (PDB code 4HSA) is shown as light blue ribbon/surface.

To understand the critical interactions of these compounds to IL17A and confirm ligand binding to a location proximal to Met110, we solved an initial crystal structure of Cmpd 6 (Fig. 3A) at 1.94 Å resolution. Interestingly, two very well-defined copies of the same ligand were identified in a pocket distinct from the primary IL17A-IL17RA interaction site. Both molecules were bound proximally to Met110 thereby confirming the previously-mentioned NMR observations. In the presence of Cmpd 6, the lack of symmetry-breaking perturbations in the NMR spectrum of the IL17A homodimer imply that both sites could be occupied simultaneously in solution. Extensive water molecules were modeled in the x-ray structure, which also played an important role in this region of the protein. Importantly, the two ligands spanned the pocket in a symmetrical fashion, making interactions with identical residues from both monomers in the IL17A homodimer. The protein–ligand interactions we observed were: (1) hydrogen bonding from the ligand N-linkers to the backbone carbonyls of Ile100; (2) central pyridyls hydrogen bonded to a solvent network; (3) Van der Waals interactions of the quinoline moieties in a secondary pocket proximal to the sidechain of Ile100 and the protein backbone of nearby residues**.** In these initial structures (and became subsequently relevant), His109 was not engaged in this co-crystal structure.

Similarly, the structure of Cmpd 5 was solved to 2.28 Å resolution with similar observations: two ligands in the “Methionine” binding pocket and interactions between the linking nitrogens to the backbone carbonyls of Ile100 (Fig. 3B). The terminal morpholine group emerged toward the outside of the pocket. It was modeled and then subsequently refined in relatively weak electron density. Both the Cmpd 5 and Cmpd 6 structures show a fair amount of polar negative charge as seen from the generated electrostatic surfaces. One significant change observed between these structures was that the side chain of the C-terminal residue His152 in Cmpd 5 moved by 8 Å in one IL17 monomer to make a direct contact with the pyridyl N of the ligand while making a more “extended” conformation in the structure with Cmpd 6 (Fig. 3C,D). This demonstrated the dynamic nature of this region of IL17A, and we note that some unassigned valine and leucine peaks shifted upon binding that were presumably near Met110. Overall these observations contributed to an emerging hypothesis that the C-terminal domain had conformational flexibility, which could be important in its association with IL17R.

When examining the previously solved structure of the IL17A/IL17RA complex (PDB code 4HSA), the C-terminal strand of the IL17A/A homodimer is involved in the association of the D2 domain of the receptor. The C-terminus acts as an additional strand to β3 of the β-sheet in this domain (Fig. 3D) and we note that proper alignment of IL17A C-terminus is necessary to create the interface between the two IL17A subunits in the homodimer. Due to the proximity of the “Methionine pocket” and the C-terminus together with the conformational changes observed in the ligand bound structure, we hypothesized that molecules bound to this region could act as potential inhibitors, affecting and disrupting the interface between IL17A and the D2 domain of IL17R such that optimized interactions could drive useful disruption of key cell signaling events. This idea is supported by the observation that the IL17A-bound structures of Cmpd 5 and Cmpd 6 in the C-terminus show different positioning in this area of the cytokine (compared to the receptor-bound structure) with potential to interrupt or alter IL17A-IL17 receptor association.

### Deletion of C-terminal site suggests region is important for binding to IL17 receptor

**Figure 4:**
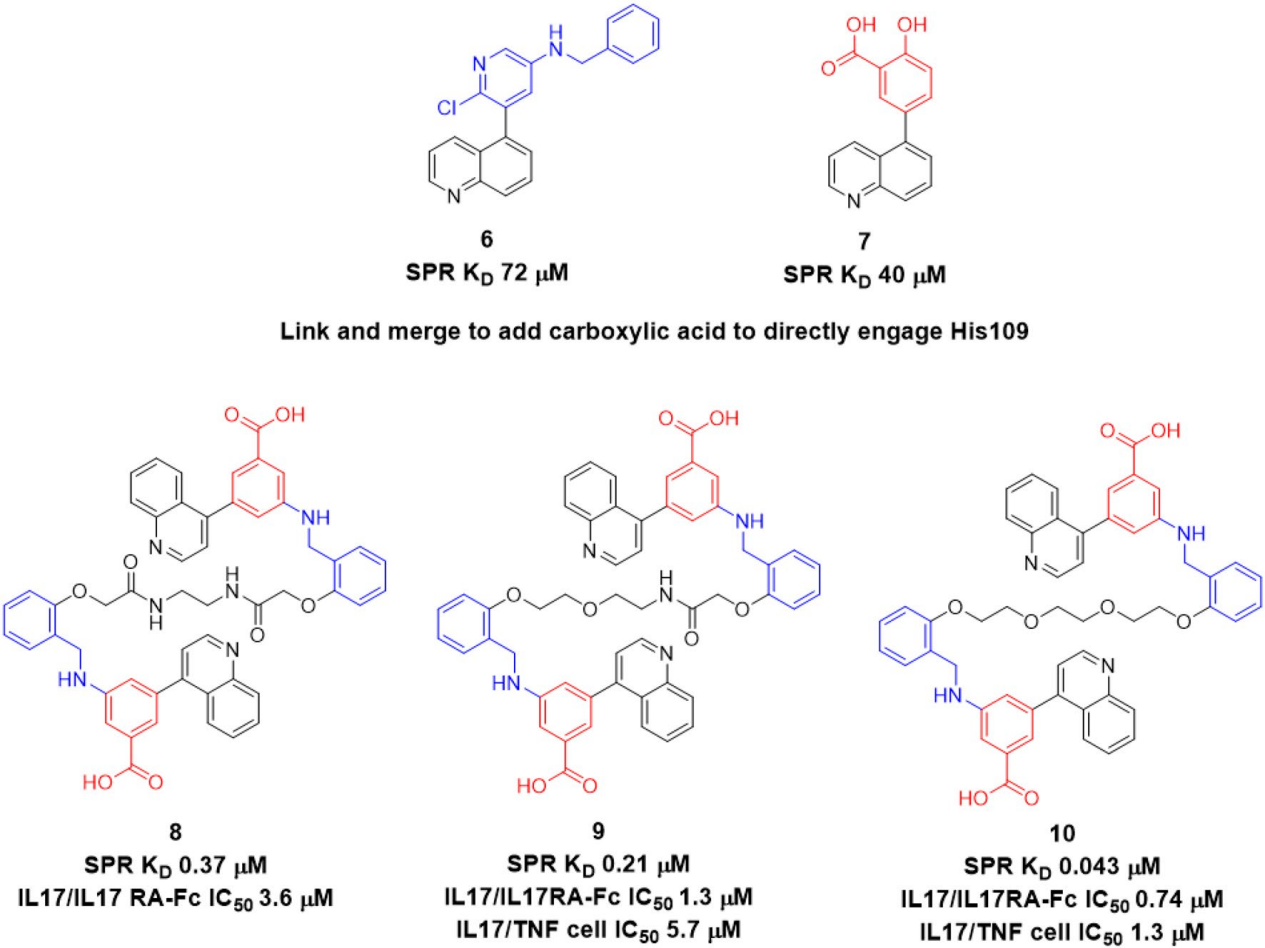
Linking strategy toward higher affinity inhibitors.

To further understand if perturbations to this region of the IL17A homodimer were likely to impact functional engagement of IL17 receptors and ultimately lead to disruption in cytokine signaling, we created an IL17A construct (aa 34–148) lacking the final six C-terminal residues (aa 149–155). In comparison to the full-length IL17A dimer, which robustly binds IL7RA as detected by SPR (*K*_D_ ~ 2 nM), we found that the C-terminal deletion did not productively interact with IL17RA (Supplemental Fig. 4). We then postulated that inhibitor binding in this region of the IL-7A cytokine might similarly abrogate binding to the IL17 receptor and subsequent signaling. With this encouraging hypothesis, we next turned to improving the affinity and structural engagement to IL17A for these early small molecule hits by using a linking strategy (Fig. 4).

### Paths to improved affinity

**Figure 5:**
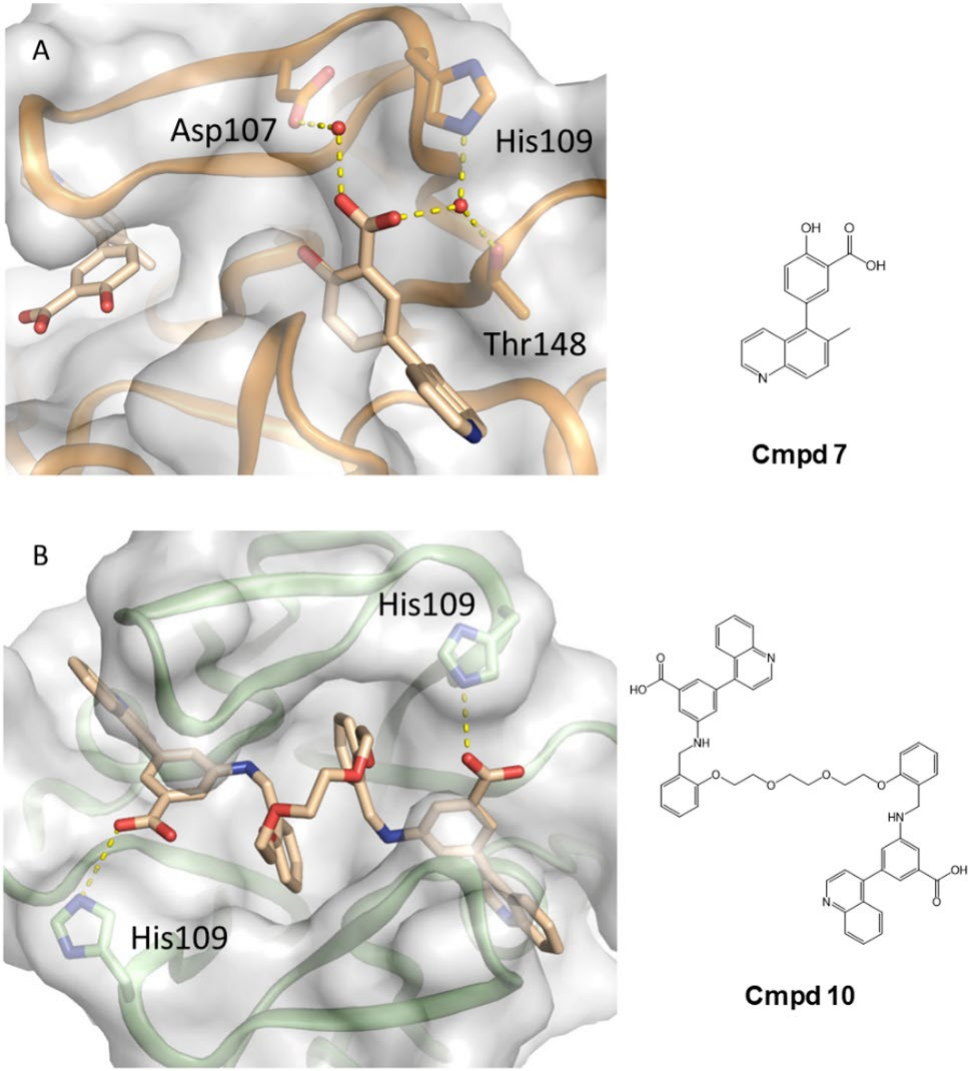
(**A**) Salicylate fragment Cmpd 7 bound to IL17A. (**B**) Structure of Cmpd 10 and highlighted interaction with His109 residues in each IL17A subunit.

In addition to Cmpd 5, we also identified a salicylate fragment (Cmpd 7) as a binder to the C-terminal site in IL17A. We solved the co-crystal structure of this compound, and it also displayed two copies (Fig. 5A) that overlaid very nicely with the position of Cmpd 6. Interestingly, one copy of Cmpd 7 was observed to make a water-mediated interaction with His109 through its carboxylic acid moeity (in the analogous position as Cmpd 6’s pyridine). That same water molecule appeared to be further stabilized by Thr148 and the carboxylic acid moiety also utilized another water molecule to engage Asp107. From the information we had uncovered, we could identify two paths to improving overall affinity. The first was to merge aspects of the quinoline fragment with salicylate fragment by swapping the central pyridyl ring for a benzoic acid to productively engage His109, Thr148, and Asp107 (as seen in the salicylate fragment, Cmpd 7). The second strategy was to link the two copies based on the hypothesis that the chelation effect could drive additional affinity^20^.

### Compounds 8–10 offer improvement in potency and show functional inhibitory properties

This strategy led to Cmpd 8, which contains both the acid substitution and an ethylene diamide linker. Our linked compound Cmpd 8 showed a dissociation constant (*K*_D_) 0.37 µM for inhibitor binding to IL17A as determined by SPR. Using a binding assay pairing labeled IL17A protein with IL17RA-Fc protein that used AlphaLISA technology to detection complex formation, we found that Cmpd 8 could disrupt formation of the IL17A-IL17RA-Fc complex with an IC_50_ of 3.6 µM (Supplemental Fig. 5). Notably, the TruHits counter screen we used to eliminate non-specific effects such as compound-dependent absorbance and other trivial effects that could potentially give false inhibitory signals in AlphaLISA demonstrated a larger IC_50_ (~ 54 µM), indicating specific on-target effects with our IL17A-targeted inhibitor in the low µM range. Though we do not completely understand the ~ 10X upshift in IC_50_ values in the AlphaLISA relative to SPR affinity we observed with these molecules, it is most likely due to effects of including the IL17RA-Fc (receptor) binding component acting competitively receptor binding component. We therefore obtained an improvement in affinity and subsequently showed that the new molecule could block IL17A receptor interactions as detected by SPR (Supplemental Fig. 6). Despite those favorable observations, we expected a larger improvement in affinity/potency by bringing two moieties into a single molecule under an ideal binding configuration than what we observed in this instance.

**Figure 6:**
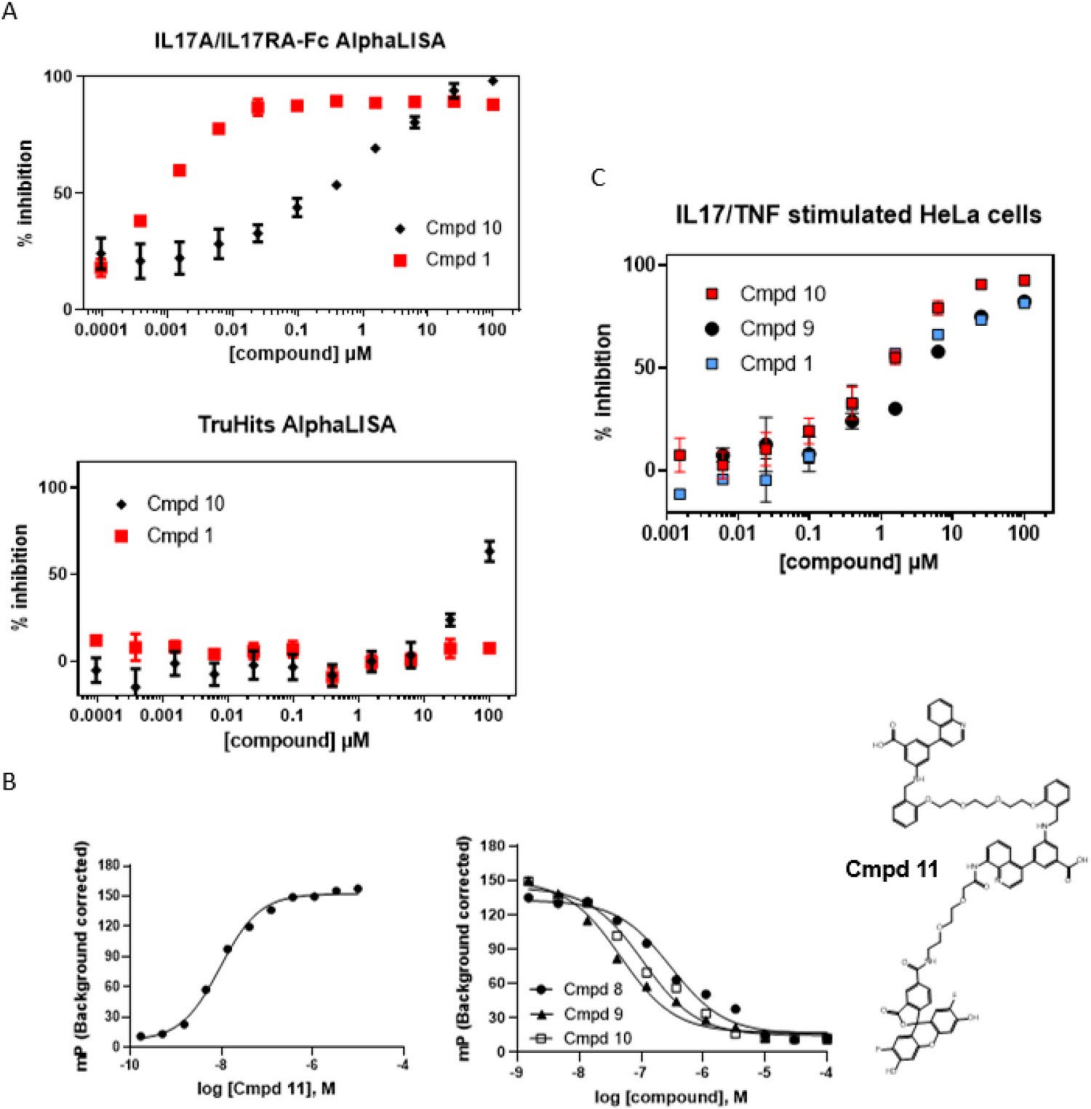
Biochemical and cellular data for IL17A inhibitors (**A**) Biochemical interaction assay (**B**) FP probe for C-terminal site occupancy: (left) titration of fluoroprobe, Cmpd 11; (middle) competition assay with unlabeled inhibitors and (right) structure of Cmpd 11 (**C**) Cellular inhibition of IL17 signaling. Error bars shown in (**A**,**C**) reflect the standard error of the mean.

Thus, the next stage in our optimization was to eliminate potential negative strain in the linker upon binding. To accomplish this, we kept the same length of linker from Cmpd 8 and swapped the amide for an ethylene glycol, resulting in Cmpd 9 (SPR *K*_D_ 209 nM; IL17A/IL17RA-Fc AlphaLISA IC_50_ of 1.3 µM with TruHits counterscreen IC_50_ of ~ 57 µM; Supplemental Fig. 5B). While the region of the molecule with the amide intact was expected to interact similarly as Cmpd 8, the region of the molecule with the ethylene glycol linker could also make a direct H-bond interaction with His107 of monomer 2. To recapitulate this direct interaction on the other side of the molecule, we next substituted the other amide in the linker with an ethylene glycol to make Cmpd 10. This compound gave us an X-ray co-crystal structure as seen in Fig. 5B, where no major changes in protein structure/gross rearrangements were observed. Isothermal titration calorimetry experiments with Cmpd 10 showed stoichiometry consistent one molecule bound per IL17A homodimer, where it gave affinity of 44 nM (Supplemental Fig. 7). Cmpd 10 also showed increased SPR affinity (43 nM) relative to Cmpd 9 and showed inhibition of IL17A/IL7RA-Fc interactions (IC_50_ 740 nM: Fig. 6A). This compound was further leveraged to make a fluorescence polarization (FP) probe (Cmpd 11) (Fig. 6B), Using this probe, Cmpd 10 gave an IC_50_ of 107 nM in FP assays when the unlabeled compound was pre-bound to IL17A before addition of FP probe. Cmpd 8 and Cmpd 9 also showed full competition for IL17A with this probe (Fig. 6B).

We next tested our compounds in an IL17-dependent cell-based assay in which recombinant TNF and IL17A stimulated HeLa cells carrying an LCN2 promoter-driven luciferase reporter. Those studies showed that Cmpd 8 blocked IL17A/TNF stimulated cells with IC_50_ 4.7 µM while having IC_50_ ~ 51 µM if the same cells were stimulated only with TNF. The increased potency in the presence of IL17A suggested engagement of IL17A was driving stronger effects via specific targeting. When we examined subsequent compounds, we found that Cmpd 9 inhibited IL17A/TNF stimulated cells with IC_50_ 5.7 µM (Fig. 6C), while TNF-only stimulated HeLa reporter cells gave an IC_50_ > 70 µM. Cmpd 10 inhibited IL17A/TNF stimulated cells with IC_50_ 1.3 µM (Fig. 6C), compared to a much weaker TNF-only stimulated IC_50_ > 50 µM. Thus our drug discovery efforts at this C-terminal site yielded IL17 selective, cell-active molecules with moderate potency.

## Discussion

Interleukin 17 has been shown to drive pathogenic effects in numerous inflammatory disorders across disease areas such as rheumatology, dermatology and other conditions associated with excess immune responses^8,22^. For example, the action of IL17A has been shown to be central to the development of skin inflammation in psoriasis and, indeed, several biologics targeting the cytokine can greatly improve the symptoms of psoriasis in large percentages of patients^23,24^. Orally available IL17A small molecule antagonists could extend these benefits to a larger number of patients who dislike the needle-based injections needed to administer such anti-IL17 antibodies or need to more quickly wash out IL17 antagonism in the event of fungal or other infections. To this end, we and others have been interested in finding suitable binding sites on the IL17A homodimer where small molecule inhibitor binding can functionally disrupt IL17A signaling.

As described here, we were able to utilize NMR based fragment screen (Fig. 2) together with rational structure-based drug design to identify a previously undescribed binding mode for IL17 small molecules interacting at the C-terminus of the IL17A/A homodimer (Fig. 3). This novel site was first indicated by the disturbance of the NMR resonance of a Met residue near the C-terminus (Met110), then confirmed by mutagenesis studies and x-ray structures. Through inspection of the published IL17A/IL17 receptor A x-ray co-crystal structure^21^ we noted that this area of the cytokine made significant contacts to engage its cognate receptor. Moreover, through deletion of this C-terminal region in IL17A, we found that this area imparted important contributions to IL17 receptor binding (Supp Fig. 4), furthering the notion that disrupting interactions in this location could potentially block IL17 signaling.

Starting with a fragment hit (Cmpd 3) that could alter Met110 chemical shift in NMR (Fig. 2D) but had only weak initial affinity (*K*_D_ > 250 µM), we screened related analogs to identify additional binders by NMR and SPR. Two of the analogs tested, Cmpd 5 and 6, yielded x-ray co-crystal structures bound to IL17A. These revealed the critical information that two copies could bind symmetrically in a region proximal to Met110 (Fig. 3). From this observation and by comparison to another salicylate fragment (Cmpd 7) that we found also bound in this region, we were able to “link” the two copies into a larger molecule. This inhibitor bound with increased affinity (Cmpd 8; *K*_D_ 370 nM) that could specifically prevent IL17A from binding one of its receptors (IL17RA) in AlphaLISA proximity assay (IC_50_ ~ 4 µM, Supplemental Fig. 5) and also showed disruption of IL17A-IL17RA interactions in SPR-based assay (Supplemental Fig. 6). Subsequent molecules further increased affinity to IL17A (*K*_D_ < 100 nM for Cmpd 10) and importantly could also block signaling of IL17A in cellular assays (IC_50_ 1.3 µM for Cmpd 10).

This example of NMR fragment screening coupled with SPR binding analysis and x-ray crystallography highlights how new modes of inhibiting key protein–protein interactions (PPIs) can be discovered. We also report here a suitable probe molecule (Fig. 6B) that can be used by fluorescence polarization to assess if other molecules bind at this novel site.

Additional molecules binding IL17A in other locations on the homodimer structure have been previously reported. For example, Liu and colleagues at Pfizer^16^ reported a macrocycle that bound at the interface of the two IL17A monomers in a region that is also known to engage with the IL17 receptor but this region is many angstroms (Fig. 2) from the C-terminal location where our molecules bind. Notably, these molecules showed ability to block IL17A binding to IL17RA in FRET assay (IC_50_~9 nM) but gave reduced effects in cell-based assays (IC_50_~400 nM).

A related macrocycle from Ensemble and colleagues has also been described^15^ that we used a positive control for some of our biochemical and cellular assays (Fig. 6A,C). Interestingly while it brings low nM potency in biochemical assays it cannot block cellular signaling until is it given at much higher concentrations (~ 3 µM). The reasons that molecules like Cmpds 1 and 2 show larger potency shifts between biochemical assays & cell-based signaling assays are unclear are present. We speculate this might be related to binding at sites mostly along a single receptor-binding interface in the homodimeric IL17A protein as evident in the Pfizer macrocycle x-ray structure. Since IL17A has a second symmetry-related receptor binding site opposite this, leaving this other region of the molecule unoccupied may be a disadvantage to preventing cell-based signaling that is not revealed in simplified biochemical binding competition assays. At another very distal site on IL17A, a synthetic “high affinity peptide” binding the N-terminus of IL17A (Fig. 2) with *K*_D_ 1 nM was shown by Pfizer researchers to bind two copies to IL17A and disrupt cell signaling^18^ (IC_50_~150 nM). The challenge with these previous IL17A inhibitors as well as the molecules we describe here is to obtain suitable solubility & permeability to enable robust pharmacokinetics. A recent report from Leo Pharma shows this favorable oral pharmacokinetics for IL17A inhibitors^25^ interacting near the site on the cytokine where Cmpd 2 binds.

Additional patent applications from UCB, and others^26–28^ suggest other ways that IL17A may be engaged with small molecules. Now together with the C-terminal site that we described here, it is becoming clear there are multiple modes that can disrupt the critical binding of interleukin IL17A to its cognate receptors and antagonize the signaling process that is pathogenic in many widespread inflammatory disorders affecting human health. We look forward to seeing which of these sites prove to be the most amenable to producing optimized drug candidates.

## Methods

### IL17A protein production

For crystallography, NMR and early SPR studies, IL17A (34–155)C129S or IL17A (34–148)C129S was cloned into modified pET32b vector with N terminal Trx-6His-tags followed by a thrombin cleavage site. For SPR studies, IL17A (24–155) was cloned into pMAL-C5X vector with N-terminal 6His-MBP tag followed by a thrombin cleavage site and C-terminal Avi tag. Proteins were expressed in Escherichia coli Shuffle T7 express strain (NEB). His-tagged proteins were purified with Ni–Sepharose 6 affinity column, and tags were cleaved with human α-thrombin (Haematologic Technologies, Inc.). Proteins were further purified with Resource S column (Cytiva) to obtain the final mature forms. The purified IL17A (34–155)C129S or IL17A (34–148)C129S proteins were shown to migrate consistent with size of homodimers relative to standards in Size Exclusion Chromatography (see Supplemental Fig. 8A). Size Exclusion Chromatography—Multi-Angle Light Scattering (SEC-MALS) analysis of IL17A (34–155)C129S further showed predicted molecular weights consistent with dimeric forms (see Supplemental Fig. 8B).

### NMR experiments

NMR spectra were acquired on either a Bruker 500 MHz AVANCE III spectrometer or a Bruker 600 MHz AVANCE III HD spectrometer, both with cryoprobes, on 60 µM protein samples in 20 mM deuterated TRIS buffer, pH 8.0, with 150 mM NaCl. For fragment screening, fragments were added from a concentrated DMSO stock to a concentration of 1 mM in the NMR tube. As described above, fragments were initially added as mixtures of 12 and those mixtures which induced significant changes in a 13C-HSQC spectrum of the protein were then deconvoluted to find the individual binders.

### Compound binding to IL17A and IL17A/IL17RA binding via surface plasmon resonance

Compounds were assayed via Surface Plasmon Resonance (SPR) using Biacore T200 instrument and Biacore 4000 [Formerly GE Healthcare (now Cytiva Life Sciences)]. Biotinylated Avi-tag IL17A-(24–155) was produced by enzymatic biotinylation and subsequently captured on immobilized neutravidin surface, where multi-cycle kinetics and single-cycle kinetics modes were used. The compounds were diluted in the running buffer (10 mM Hepes, pH 7.5, 150 mM NaCl, 0.005% Tween-20 and 3% DMSO) and injected in a series of increasing concentrations at flow rate of 100 µL/min for contact time of 60 s (for weak compounds) or 200 s (for potent compounds), and dissociation was monitored for up to 500 s (for weak compounds) or 8000 s (for potent compounds). Sensorgrams were processed (including solvent correction) and analyzed using Biacore T200 and Biacore 4000 evaluation software. The binding curves were fit to determine the equilibrium dissociation constant (K_D_) and kinetic constants (k_on_ and k_off_).

To interrogate IL17A—IL17RA binding and the effect of compound binding to IL17A for interaction with IL17RA, Biotinylated Avi tag IL17RA was immobilized using CAP chip. IL17A WT protein with and without compounds was injected over the immobilized IL17RA surface. The surface was regenerated using mixture of 8 M Guanidine Hydrochloride and 1 M Sodium Hydroxide. To profile both IL17A WT and truncated-IL17A binding to IL17RA, Fc tag IL17RA (Sino Biological) was captured on immobilized anti-Fc (Human Fc from Pierce) surface. IL17A WT (34–155)C129S and Truncated (34–148)C129S was injected at flow rate of 80 µL/min for contact time of 180 s and dissociation was monitored for 1200 s. The surface was regenerated with 2 consecutive injections (60 s and 10 s at 60 µL/min) of 10 mM Glycine, pH 1.5.

### X-ray crystallography

IL17A protein purified as previously described was concentrated to 9.5 mg/mL in 0.1 M NaCl, 0.02 M Tris, pH 8.0. To crystallize IL17A protein with the compounds described in this paper, the compounds were first dissolved in 100% DMSO solution to a final concentration of 100 mM. Then, 2 µL of compound stock was mixed with 100 µL of protein sample to make a mixture with compound to protein molar ratio of ~ 2:1. The mixture was incubated at room temperature for 1 h and spun down before setting up crystallization trays. Sitting drop or hanging drop vapor diffusion methods were used, adding equal amount of each of protein/compound complex and precipitant solution. For Cmpd 5, Cmpd 6 and Cmpd 10 samples, small rod shape crystals were obtained after 3–5 days of incubation at 17 °C with the precipitant solution of 3.2 M NaCl, 0.1 M Tris, pH 8.5. The crystals were harvested using 100% paratone-N oil or 15% Glycerol as cryo-protectant and were flash frozen in liquid nitrogen. For Cmpd 7 sample, crystals grew under 1.6–2.0 M Sodium Formate, 0.1 M Tris, pH 7.8–8.8. 10% Glycerol was the optimal cryo-protectant solution when crystals were later flash frozen in liquid nitrogen. All diffraction data were collected at IMCA-CAT beam line of 17-ID at Argonne National Laboratory.

The data were processed using the program AUTOPROC from Global Phasing^29^. The x-ray diffraction data are summarized in Table 1. Maximum likelihood molecular replacement solutions for all co-crystal structures were determined using the program PHASER^30^ and with a preliminary human IL17 search model from the previously reported structure (Protein Data Bank entry: 4HR9)^21^. Resulting solutions were initially refined using the program REFMAC^31^ and then in the program BUSTER^32^. The program COOT^33^ was used to examine 2Fo-Fc and Fo-Fc electron-density maps and enable iterative model building. A repetitive protein flexible loop segment was found to be disordered in all 4 structures and therefore it was not modeled (Cmpd 5: Leu49-Pro60(A)/Val47-Ser63(B), Cmpd 6: Leu49-Pro60(A)/Val47-Ser63(B), Cmpd 7: Asn48-Pro60(A)/Asn48-Asp65(B), Cmpd 10: Thr56-Ser64(A)/Leu49-Thr58(B)). Ligands were fit into Fo-Fc difference density using the program AFITT^34^ with the creation of associated ligand dictionaries. Example electron density maps showing the ligands are shown in Supplemental Fig. 9. Final rounds of refinement concluded with the addition of water molecules again using BUSTER and the program PHENIX^35^. Final refinement statistics are listed in Table 1.

**Table 1:** X-ray data and structure refinement statistics.

Structure	IL17A complexed to Cmpd 5	IL17A complexed to Cmpd 6	IL17A complexed to Cmpd 7	IL17A complexed to Cmpd 10
PDB code	8DYI	8DYH	8DYG	8DYF
Data collection				
Resolution (Å)	42.2–2.3	45.3–1.9	42.3–1.5	83.4–2.0
Space Group	C2	C2	C2	P65
Unit Cell (a, b, c; Å)	172.9	170.5	170.0	96.3
	30.5	35.7	35.8	96.3
	50.2	45.7	45.5	51.9
	α = 90°, β = 102.7°, γ = 90°	α = 90°, β = 97.8°, γ = 90°	α = 90°, β = 98.2°, γ = 90°	α = 90°, β = 90°, γ = 120°
Unique reflections	12,051	19,678	40,316	18,098
Overall statistics (highest shell)				
R_sym_ (%)	0.11(0.66)	0.12 (0.70)	0.05 (0.60)	0.09 (1.3)
I/σ_I_	8.4(2.2)	6.8 (1.9)	14.3 (2.0)	16.7 (2.2)
Data completeness (%)	97.9(91.1)	82.2 (66.5)	90.0 (75.0)	100(100)
CC(1/2)	0.99(0.80)	0.99 (0.62)	1.0 (0.64)	1.0 (0.87)
Mean multiplicity	3.2(3.4)	3.1(2.8)	3.6 (3.1)	10.0 (10.3)
Refinement				
Reflections used in refinement	11,737	19,666	40,284	18,065
R_cryst_ (%)	27.2	21.2	21.1	21.4
R_free_ (%)	31.1	24.8	23.3	26.0
R.m.s. deviations, bond lengths (Å), bond angles (°)	0.008, 1.16	0.008, 1.08	0.006, 1.01	0.007, 1.13
Ramachandran statistics				
Outliers (%):	0.00	0.00	0.00	0.00
Allowed (%):	1.7	1.23	0.6	1.6
Favored (%):	98.3	97.7	99.4%	98.4

### IL17A-IL17RA-Fc AlphaLISA assay

AlphaLISA technology was used to understand the ability of compounds to inhibit IL17A binding to IL17 receptor (IL17RA). Compounds of interest were diluted in 100% DMSO and plated in a 384 well white proxiplate (Perkin Elmer) using Echo Acoustic Liquid Handler (LabCyte Sunnydale, CA). To the compound plate, 4 µL of biotinylated IL17A (R&D systems) and 2 µL of IL17RA-Fc (R&D Systems) was added, to provide a final concentration of 1 nM of IL17RA-Fc and IL17A, respectively. The assay was carried out in 100 mM Tris, 0.01% Tween-20, 0.1% BSA, pH 8.0. The assay mixture was allowed to incubate at room temperature for 60 min, after which, 2 µL of protein A AlphaLISA acceptor beads (#AL101M, PerkinElmer) was added to a final concentration of 7.5 µg/mL for 60 min. To the plate, 2 µL of streptavidin donor beads (#6,760,002, PerkinElmer) was added to a final concentration of 7.5 µg/mL. The plate was allowed to incubate for one hour at room temperature, then loaded into the Envision multimode plate reader (Perkin Elmer). AlphaLISA TruHits Kits (#AL900D, PerkinElmer) were used to assess potential false positives. All inhibition experiments were normalized against DMSO control containing no inhibitor. Example raw data are shown in Supplementary Fig. 10A. Data analyzed and reported as IC_50_s were typically from 2 to 4 replicates per inhibitor concentration.

### IL17A stimulated HeLa-LCN2 reporter cells

The promoter of the IL17 target gene lipocalin 2 (LCN2) inserted into pGL4-luciferase vector (pGL4[luc2P/LCN2/Hygro], obtained from Promega) was used to generate a stable clone in HeLa cells (ATCC) using hygromycin. In response to IL17 stimulation the increase in LCN2-luciferase gene transcription can be monitored using the luciferase reporter assay as described below. The reporter cells (HeLa-LCN2) were cultured in Dulbecco's modified Eagle's medium supplemented with 10% (v/v) fetal bovine serum, 0.2 mg/mL hygromycin, and 1% penicillin and streptomycin. Cells were maintained at 37 °C in a humidified atmosphere of 5% CO_2_. The assay media contained Dulbecco's modified Eagle's medium supplemented with 0.5% Charcoal-stripped FBS and 1% penicillin and streptomycin. About 7500 cells per well were plated in 40 ul assay media in a 384-well black plate (Grenier, #781,090) for 16 h. Then cells were stimulated with IL17A (10 ng/mL) and TNF (20 ng/mL) for 6 h at 37 °C and 5% CO2. The supernatant was removed and 25 µL of One-Glo reagent (#E6110, Promega) was added to the wells and luminescence was measured using Envision multimode plate reader. Background signal was measured using the same protocol by stimulating cells with TNF (20 ng/mL) in the absence of IL17A. All inhibition experiments were normalized against DMSO control containing no inhibitor. Example raw data are shown in Supplementary Fig. 10B. Data analyzed and reported as IC_50_s were typically from 2 to 4 replicates per inhibitor concentration.

### Fluorescence polarization assay

Fluorescence polarization (FP) assays were performed in black shallow 384-well micro plates (ProxiPlate – 384 F Plus, PerkinElmer) using Envision multimode plate reader (Perkin-Elmer). Polarization was measured by using an excitation filter of 485 nm and an emission filter of 535 nm. Each well was flashed 50 times, and the average values were used. Briefly, 5 µL of IL17A (20 nM) diluted in the assay buffer (50 mM Tris pH 8.0, 0.001% Tween) was added to compound containing wells and incubated for 60 min. To this, 5 µL of the FP probe (10 nM) was added and the reaction mixture was incubated for 120 min at room temperature prior to measuring FP. All inhibition experiments were normalized against DMSO control containing no inhibitor.

## Supplementary Information

Supplementary Information.

## Data Availability

The atomic coordinates and structure factors have been deposited in the Protein Data Bank with the primary accession codes 8DYI, 8DYH, 8DYG and 8DYF. The data supporting the findings of this study are available from the corresponding author upon reasonable request.
