# Supplementary material for: Identification and structure-based drug design of cell-active inhibitors of interleukin 17A at a novel C-terminal site
Source: Sci Rep. 2022 Aug 26;12:14561. doi: 10.1038/s41598-022-18760-1 (PMC9418147; doi:10.1038/s41598-022-18760-1)
Supplement: Supplementary file 1 — Supplementary Information. [file 41598_2022_18760_MOESM1_ESM.docx]

**Supplemental Figures**


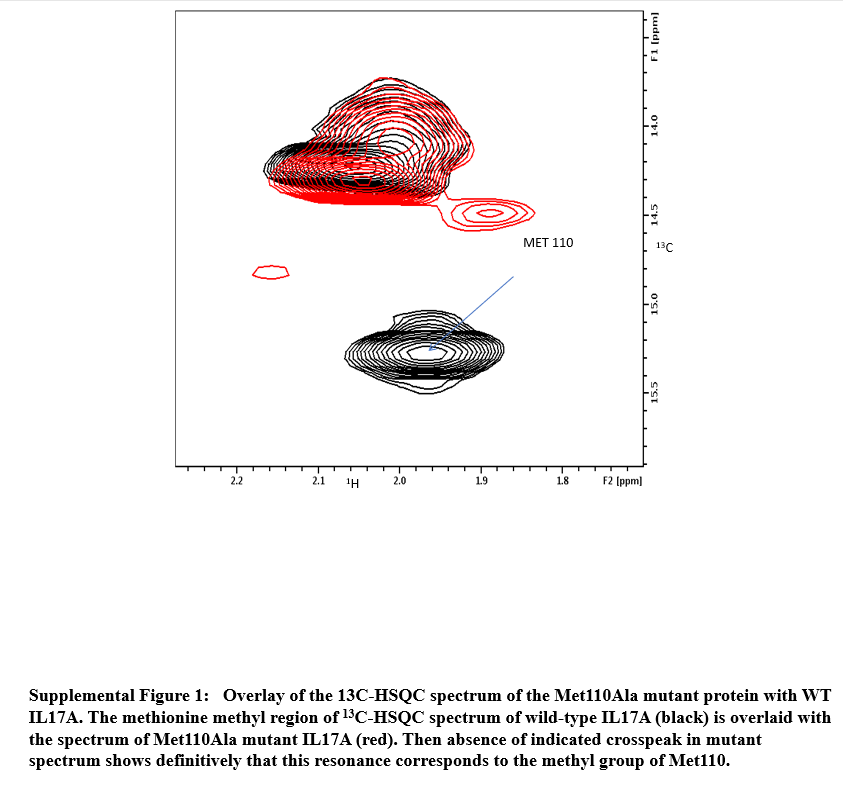


**Supplemental Figure 1: Overlay of the 13C-HSQC spectrum of Met110Ala mutant protein with WT IL17A. The methionine methyl region of ^13^C-HSQC spectrum of wild-type IL17A (black) is overlaid with the spectrum of Met110Ala mutant IL17A (red). Then absence of indicated crosspeak in mutant spectrum shows definitively that this resonance corresponds to the methyl group of Met110.**


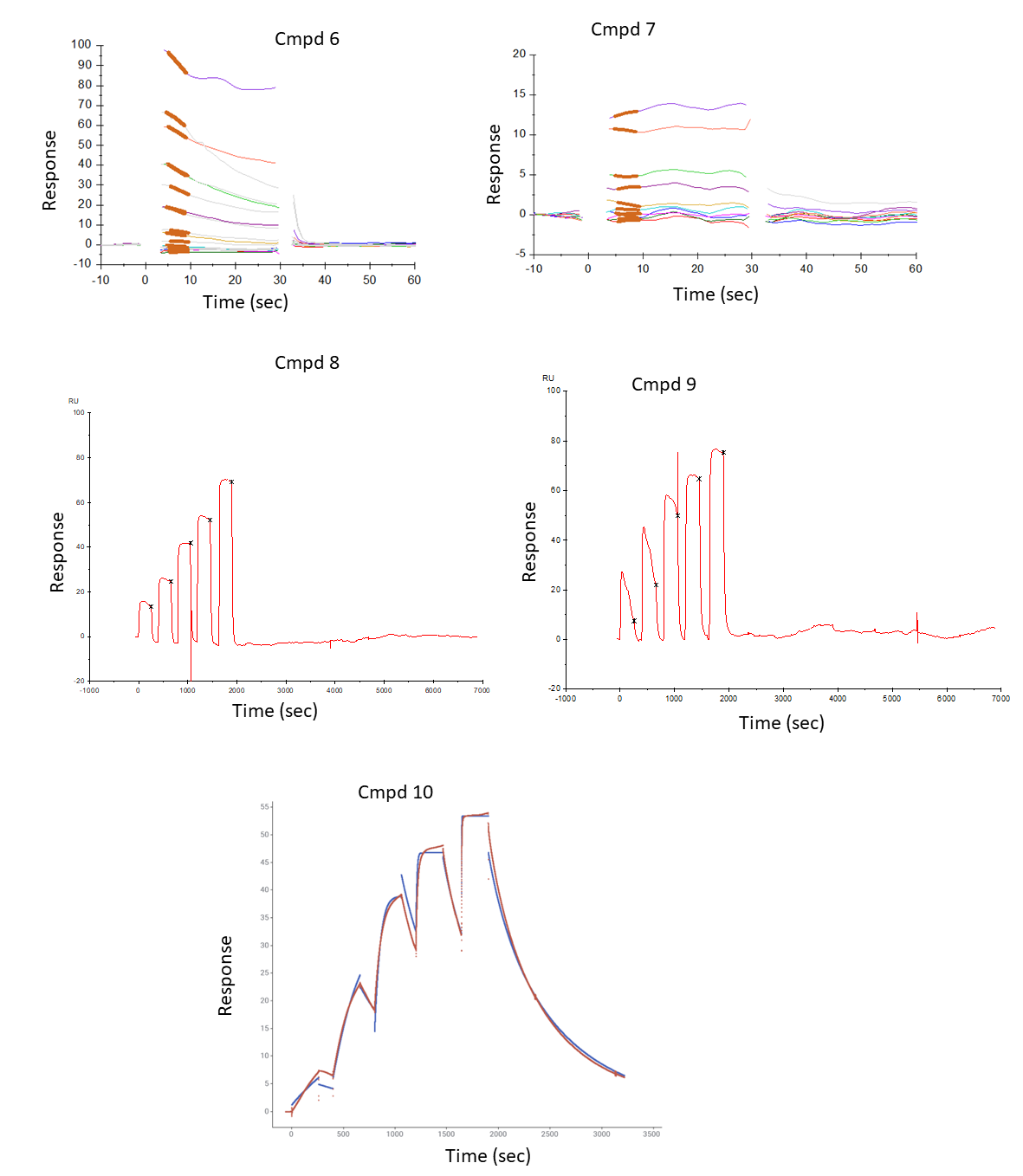


**Supplemental Figure 2: Measurement for binding of IL17A inhibitors by Surface Plasmon Resonance (SPR). Compounds 6 and 7 were profiled in multi-cycle kinetics mode; KD was determined using equilibrium responses. Compounds 8, 9 and 10 were profiled in single-cycle kinetics mode; *K*_D_ was determined using kinetic response from five concentrations of compound binding to protein.**

**Binding parameters determined are shown below.**

**
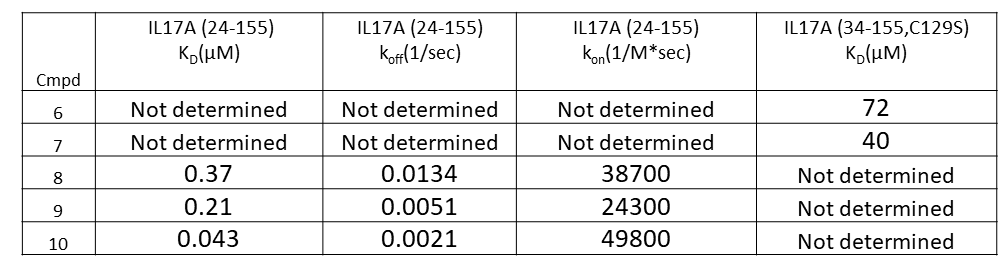
**


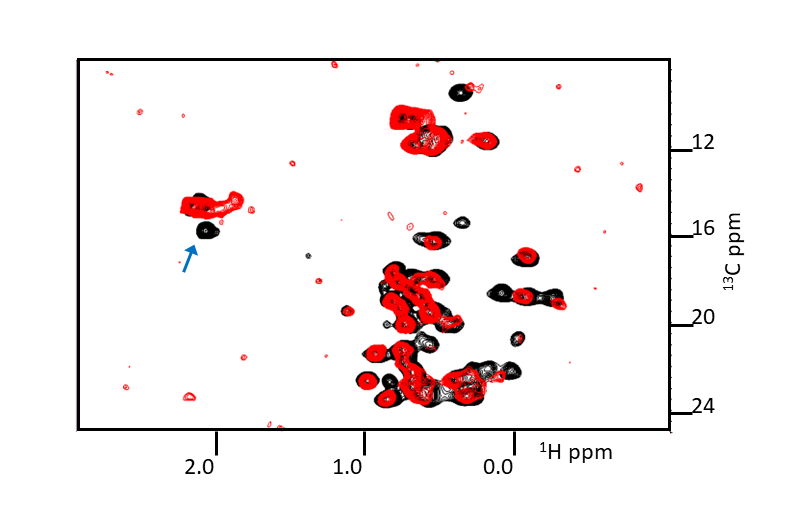


**Supplemental Figure 3: Methyl region of ^13^C-HSQC spectrum of wild-type IL17 (black) overlaid with spectrum in the presence of Cmpd 6 (red). Methyl resonance of M110 (blue arrow) is completely broadened out in the presence of the ligand. In addition, other perturbations are observed with this larger ligand as compared with those observed with the smaller fragment (Figure 2).**


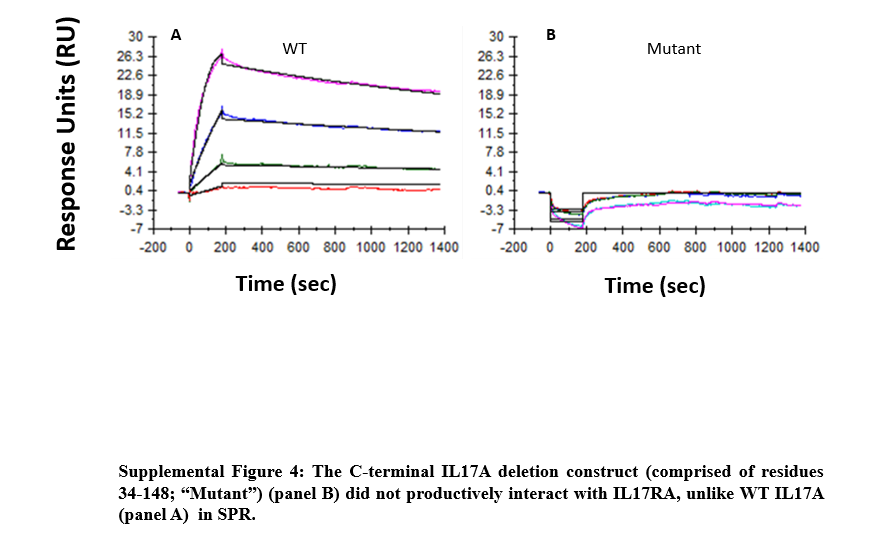


**Supplemental Figure 4: The C-terminal IL17A deletion construct (comprised of residues 34-148; “Mutant”) (panel B) did not productively interact with IL17RA, unlike WT IL17A (panel A) in SPR.**


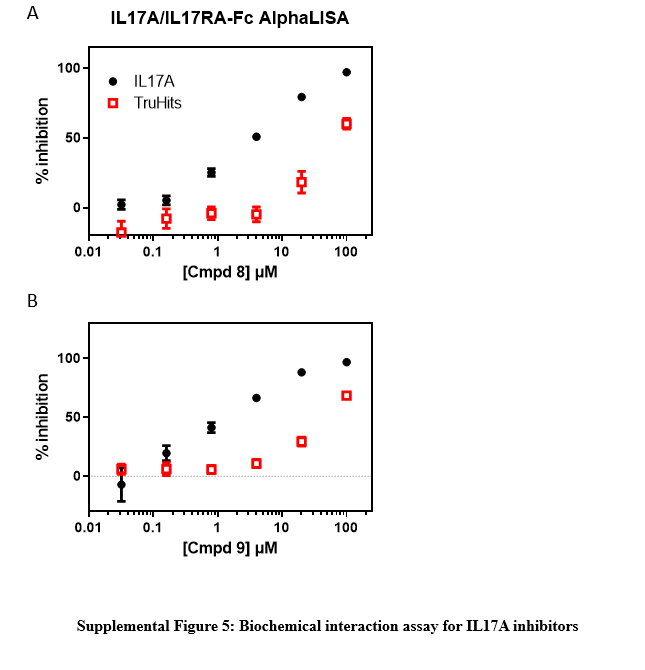
**Supplemental Figure 5: Biochemical interaction assay for IL17A inhibitors. Error bars shown reflect the standard error of the mean.**


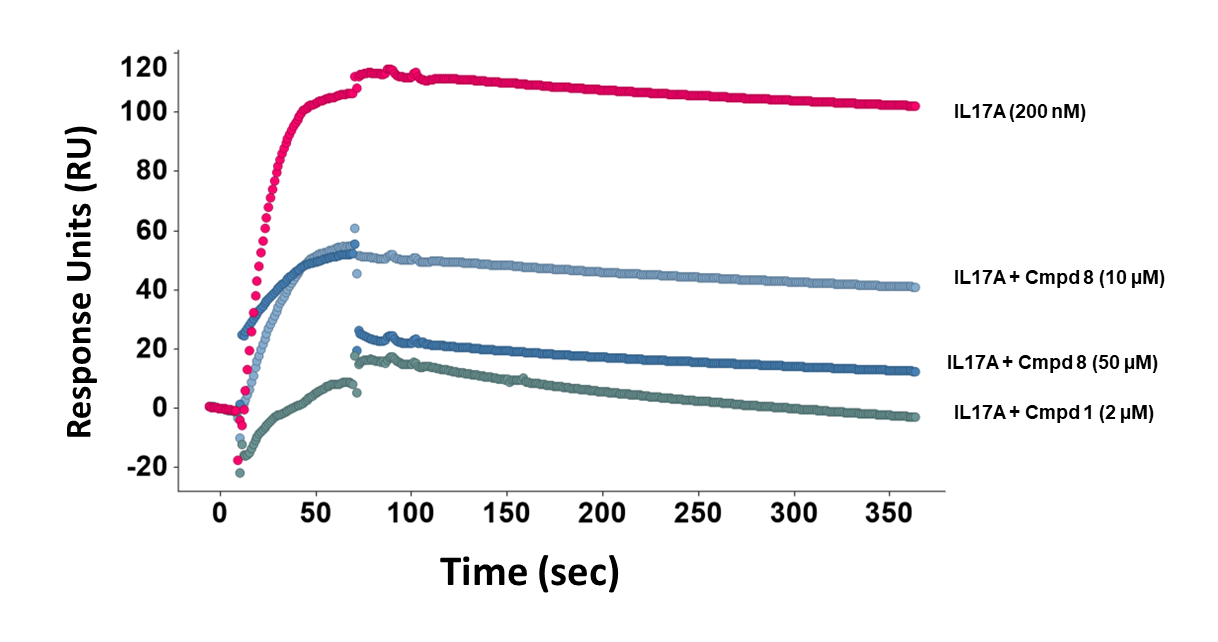


**Supplemental Figure 6: C-terminal binding site compounds can disrupt IL17A binding to its receptor in SPR competition assay. Biotinylated Avi-tagged IL17RA was captured on CAP chip; IL17A +/- inhibitor samples were injected over immobilized IL17RA . Cmpd 1 was used as the positive control.**

*K*_D_ = 44 ± 19 nM

**Supplemental Figure 7: Isothermal titration calorimetry of Cmpd 10. Theoretical binding of 1 molecule to a IL17A dimer would result in N of 0.5. Observed stoichiometry was 0.7. 200 µM IL17A (dimer) used injected (reverse titration) to 20 µM Cmpd 10 in 50 mM sodium phosphate pH 7.0.**

**
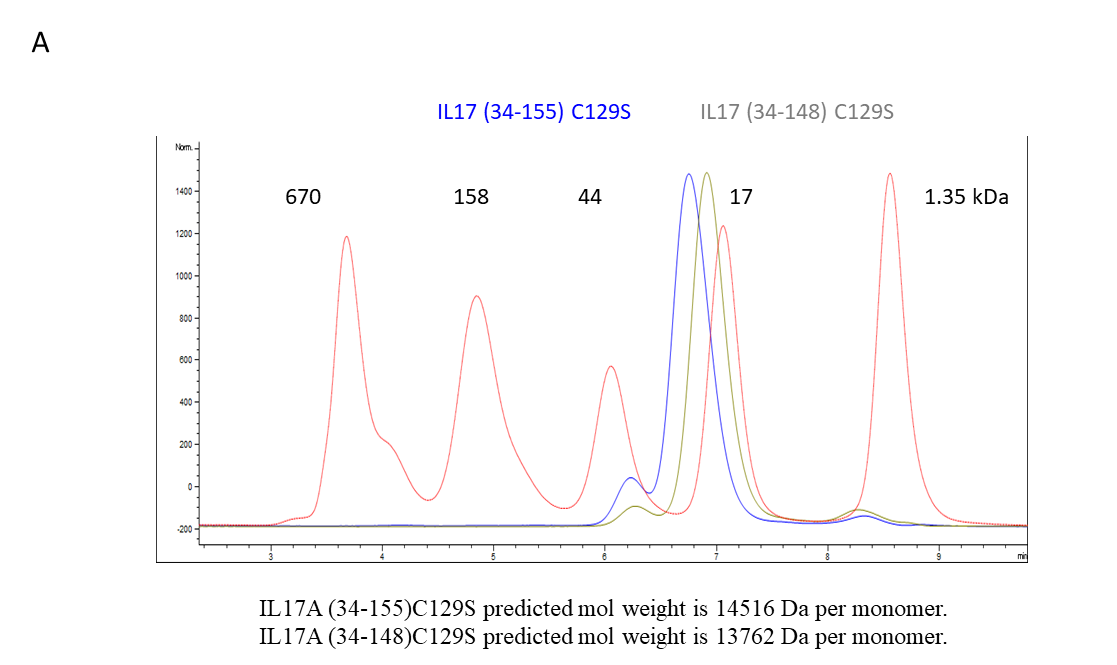
**

**Supplemental Figure 8: IL17A proteins are dimeric. (A) IL17A (34-155)C129S and IL17A (34-148)C129S proteins in Size Exclusion Chromatography relative to labeled molecular weight.**

**
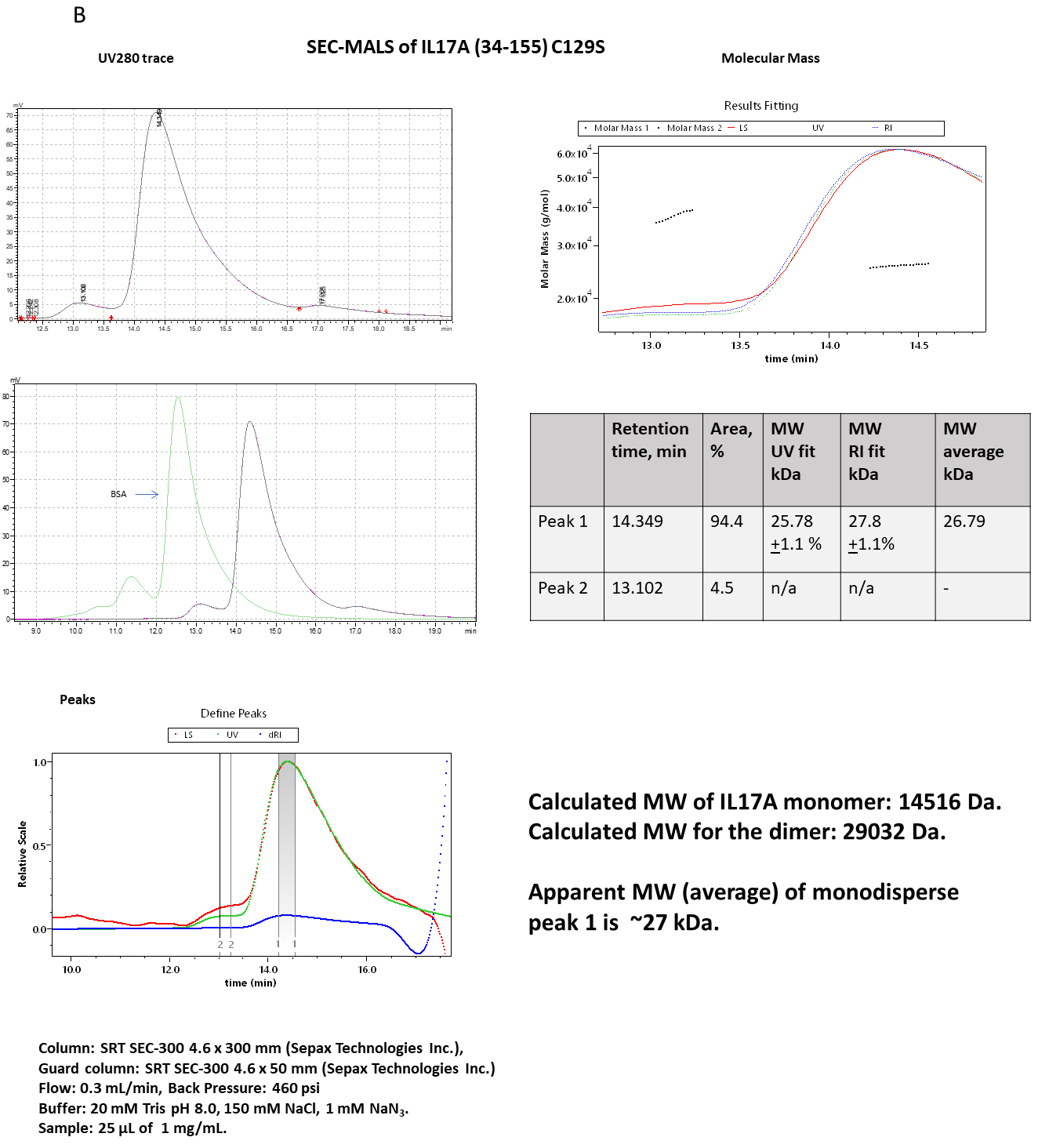
**

**Supplemental Figure 8 (cont): IL17A proteins are dimeric. (B) Size Exclusion Chromatography - Multi-Angle Light Scattering (SEC-MALS) analysis of IL17A (34-155)C129S further confirms predicted molecular weight consistent with dimeric form (see peak 1).**


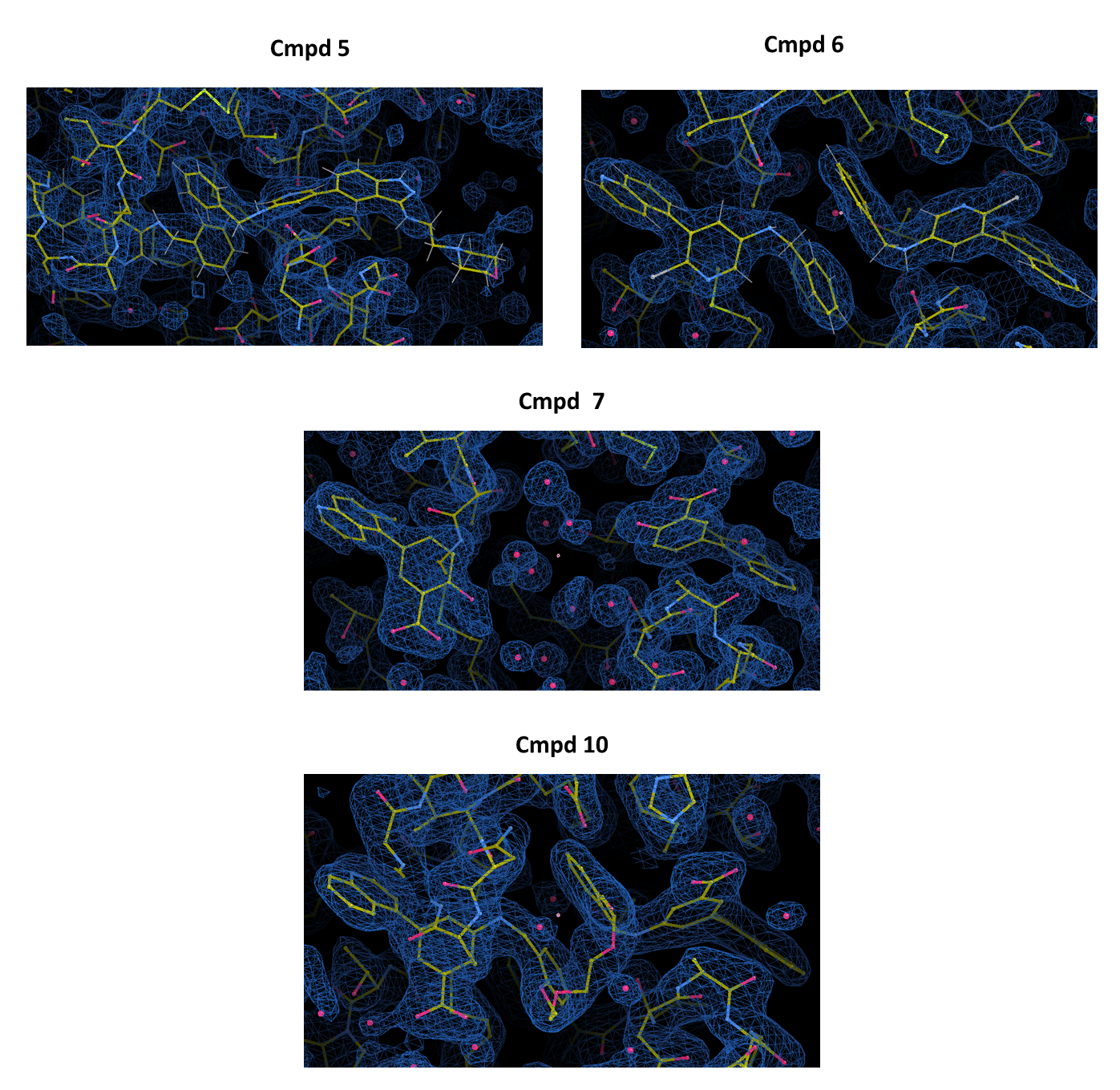


**Supplemental Fig 9: Examples electron density maps showing ligands bound to IL17A.**

**2Fo-Fc maps are shown contoured at 1σ.**


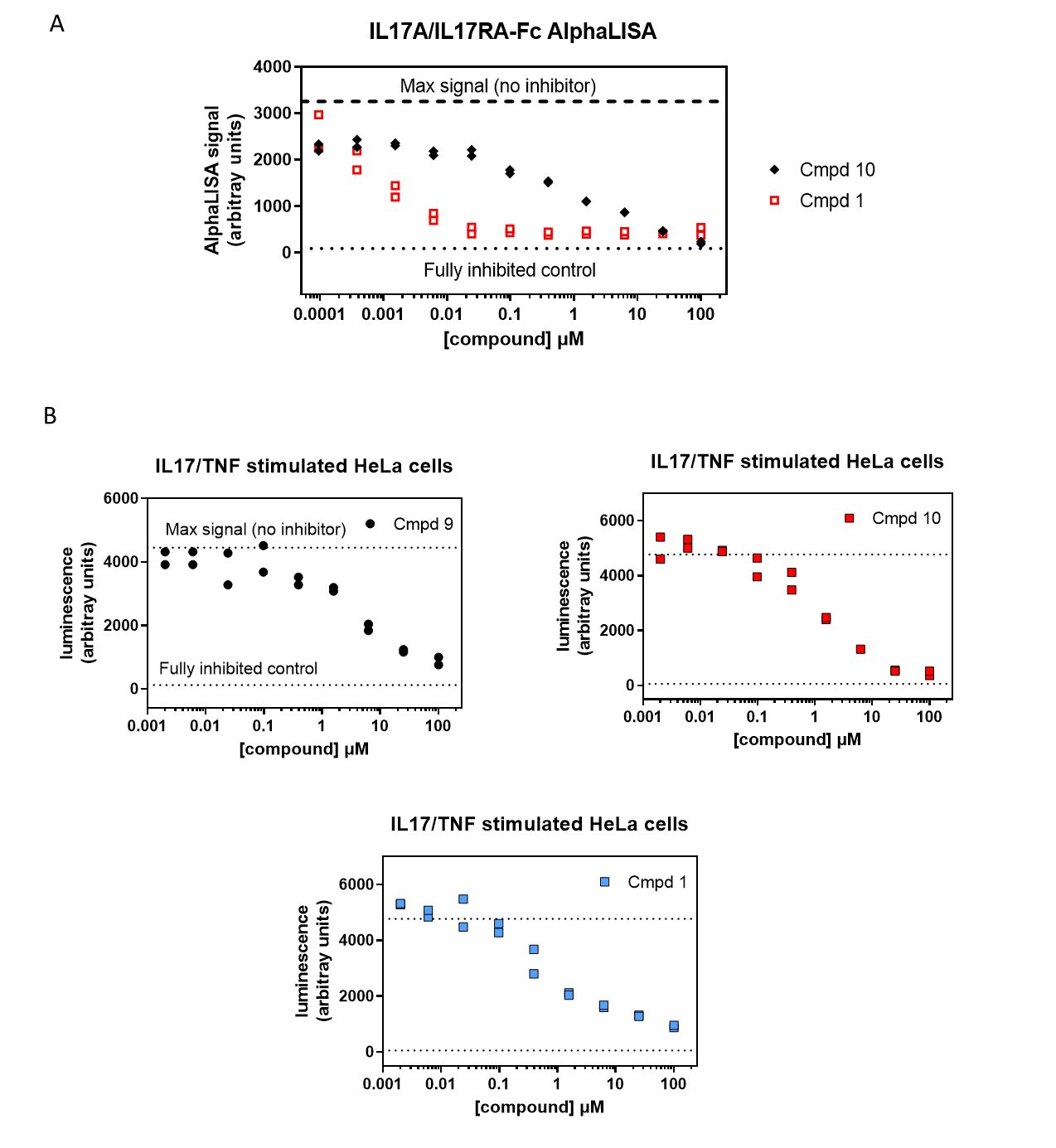


**Supplemental Figure 10: Example raw data for biochemical (A) & cellular assays (B) for IL17A inhibitors.**

Percent inhibition was calculated by using the mean maximum signal (max signal) observed in the absence of any inhibitor and the signal of a Cmpd 3 at 100 µM as fully inhibited (low control) by the equation below:

% inhibition = (1 – ((response – low control) / ( max signal – low control))) * 100

**Supplemental Methods**

**Compound synthesis methods**

**Analytical LC/MS methods**

Analytical LC-MS was performed on a Thermo MSQ-Plus mass spectrometer and Agilent 1100/1200 HPLC system running Xcalibur 2.0.7, Open-Access 1.4, and custom login software. The mass spectrometer was operated under positive APCI or ESI ionization conditions dependent on the system used.* The HPLC system comprised an Agilent Binary pump, degasser, column compartment, autosampler and diode-array detector, with a Polymer Labs ELS-2100 evaporative light-scattering detector. The column used was a Phenomenex Kinetex C8, 2.6 μm 100Å (2.1mm × 30mm), at a temperature of 65°C. A gradient of 5-100% acetonitrile (A) and 0.1% trifluoroacetic acid in water (B) was used, at a flow rate of 1.5 mL/min (0-0.05 min 5% A, 0.05-1.2 min 5-100% A, 1.2-1.4 min 100% A, 1.4-1.5 min 100-5% A. 0.25 min post-run delay).

**Synthesis of 5,5'-((((((ethane-1,2-diylbis(azanediyl))bis(2-oxoethane-2,1-diyl))bis(oxy))-bis(2,1-phenylene))bis(methylene))bis(azanediyl))bis(3-(quinolin-4-yl)benzoic acid) (Compound 8)**

**Preparation of methyl 3-amino-5-(quinolin-4-yl)benzoate:** A mixture of quinolin-4-ylboronic acid (11.28 g, 65.2 mmol), methyl 3-amino-5-bromobenzoate (15 g, 65.2 mmol), potassium acetate (12.80 g, 130 mmol), PdCl2(dppf)-CH_2_Cl_2_ adduct (2.66 g, 3.26 mmol) in 5:1 dioxane water (300 mL) was stirred at 100 ºC under nitrogen for 16 hr. The mixture was cooled, filtered, and concentrated under vacuum. The residue was dissolved in ethyl acetate (200 mL) and washed with sodium bicarbonate (200 mL) and brine (200 mL). The organic layers was collected and concentrated onto silica gel and purified by FLASH chromatography (ethyl acetate/petroleum ether, 10-50%) to yield methyl 3-amino-5-(quinolin-4-yl)benzoate (8.9 g, 31.6 mmol, 48.5 % yield) as a yellow solid. ^1^H NMR (CDCl_3_, 400 MHz): d (ppm) 8.94 (s, 1 H), 8.12 (d, J= 15.6Hz, 1 H), 7.89 (d, J =8.4 Hz, 1 H), 7.80 (m, 1 H), 7.62 (m, 1 H), 7.45 (d, J =4.4 Hz, 1 H), 7.35 (s, 1 H), 7.18 (s, 1 H), 6.95 (s, 1 H), 5.70 (s, 2 H), 3.83 (s, 3 H).

RT: 1.49 min, MS: 279 [M+1]

**Preparation of 5,5'-((((((ethane-1,2-diylbis(azanediyl))bis(2-oxoethane-2,1-diyl))bis(oxy))-bis(2,1-phenylene))bis(methylene))bis(azanediyl))bis(3-(quinolin-4-yl)benzoic acid) (Compound 8):** In an 8 mL vial with magnetic stirrer was added methyl 3-amino-5-(quinolin-4-yl)benzoate (100 mg, 0.360 mmol), N,N'-(ethane-1,2-diyl)bis(2-(2-formylphenoxy)acetamide) (69.1 mg, 0.180 mmol), and 4mL AcOH/NaOAc buffer (pH=4.6) in methanol. Siliabond Cyanoborohydride (R66730B, Silicycle) (1078 mg, 1.078 mmol) was then added and the reaction was allowed to proceed overnight. The contents were then filtered through celite, concentrated under vacuum, and then partitioned between DCM (50 mL) and saturated sodium bicarbonate (50 mL). The organic layer was dried onto silica gel and purified by flash chromatography (EtOAc/heptane, 10-100%). The purified fractions were then dried and dissolved in a solution of lithium hydroxide (1797 µl, 1.797 mmol). The reaction mixture was heated to 50 ºC for 3 hours and was then dried onto celite and purified reverse phase FLASH (10-90% MeCN/Water with 0.1% ammonium acetate) to yield the product as a white solid. ^1^H NMR (501 MHz, DMSO) δ 9.17 (d, *J* = 5.1 Hz, 2H), 8.23 (dd, *J* = 8.5, 1.0 Hz, 2H), 8.06 - 7.97 (m, 4H), 7.93 (dd, *J* = 8.6, 1.3 Hz, 2H), 7.79 (d, *J* = 5.1 Hz, 2H), 7.71 (ddd, *J* = 8.3, 6.8, 1.2 Hz, 2H), 7.46 (t, *J* = 1.9 Hz, 2H), 7.31 (dd, *J* = 7.5, 1.7 Hz, 2H), 7.28 - 7.26 (m, 2H), 7.22 (td, *J* = 7.8, 1.8 Hz, 2H), 7.01 - 6.95 (m, 4H), 6.87 (d, *J* = 8.2 Hz, 2H), 4.46 (s, 4H), 4.44 (s, 4H), 3.17 - 3.10 (m, 4H). ^13^C NMR (101 MHz, DMSO) δ 168.64, 167.93, 155.88, 149.92, 149.60, 149.18, 147.29, 138.32, 132.45, 130.91, 128.93, 128.42, 127.78, 127.70, 126.31, 125.92, 121.69, 121.53, 118.41, 117.15, 114.05, 113.86, 112.34, 67.55, 49.06, 41.85. LC/MS: RT: 0.75 min, MS: 881.4 [M+1]

**Synthesis of 3-((2-(2-(2-(2-(2-(((3-carboxy-5-(quinolin-4-yl)phenyl)amino)methyl)-phenoxy)acetamido)ethoxy)ethoxy)-benzyl)amino)-5-(quinolin-4-yl)benzoic acid (Compound 9)**

**Preparation of tert-butyl (2-(2-(2-formylphenoxy)ethoxy)ethyl)carbamate:** In a 40 mL scintillation vial with magnetic stirrer was added 2-hydroxybenzaldehyde (1.261 g, 10.32 mmol) followed by the addition of dry POTASSIUM CARBONATE (3.80 g, 27.5 mmol). The reaction was stirred at 80 deg for 15 minutes to preform the potassium salt. The reaction was cooled and 2-(2-((tert-butoxycarbonyl)amino)ethoxy)ethyl methanesulfonate (1.95 g, 6.88 mmol) was added and the reaction mixture was heated to 110 deg for 1 hour. The DMF solution was poured onto ice (100 g) and was then stirred for 60 minutes. The product separated as an oil that was extracted with ethyl acetate (100 mL). The organic layer was washed two times with LiCl (50 mL), was concentrated onto silica, and was purified via FLASH chromatography (10-100% EtOAc/Heptanes) to yield tert-butyl (2-(2-(2-formylphenoxy)ethoxy)ethyl)carbamate (1.35 g, 4.36 mmol, 63.4 % yield) as an off-white waxy solid. RT: 0.90 min, MS: 310.4 [M+1].

**Preparation of methyl 3-((2-(2-(2-aminoethoxy)ethoxy)benzyl)-amino)-5-(quinolin-4-yl)benzoate hydrochloride:** In a 50 mL round bottom flask with magnetic stirrer was added methyl 3-amino-5-(quinolin-4-yl)benzoate (1 g, 3.59 mmol), tert-butyl (2-(2-(2-formylphenoxy)ethoxy)ethyl)carbamate (1.112 g, 3.59 mmol), and 20 mL AcOH/NaOAc buffer (pH=4.6) in methanol. Siliabond CyanoborohydrideSODIUM CYANOBOROHYDRIDE (10.78 ml, 10.78 mmol) (R66730B, Silicycle) was then added and the reaction was allowed to proceed overnight. The reaction was then filtered through celite, concentrated under vacuum, and then partitioned between DCM and saturated sodium bicarbonate. The organic layer was collected, dried over magnesium sulfate (10 g), and was dried under vaccum. The crude mixture containing methyl 3-((2-(2-(2-((tert-butoxycarbonyl)amino)ethoxy)ethoxy)benzyl)amino)-5-(quinolin-4-yl)benzoate (2, 3.50 mmol, 97 % yield) boc-protected amine was dissolved in DCM (5 mL) and was added to a 20 mL scintillation vial followed by the addition of hydrochloric acid in dioxane (3 mL, 4N). This reaction proceeded for 2 h at which time LC/MS showed complete removal of Boc. Trituration via the addition of ~5 mL heptanes yielded methyl 3-((2-(2-(2-aminoethoxy)ethoxy)benzyl)-amino)-5-(quinolin-4-yl)benzoate hydrochloride (0.53 g, 1.043 mmol, 29.0 % yield) that was used directly in the next step without further purification. RT: 0.74, MS: 472.3 [M+1].

**Preparation of -((2-(2-(2-(2-(2-(((3-carboxy-5-(quinolin-4-yl)phenyl)amino)methyl)-phenoxy)acetamido)ethoxy)ethoxy)-benzyl)amino)-5-(quinolin-4-yl)benzoic acid trifluoroacetic acid salt:** In a 4 mL vial with magnetic stirrer was added methyl 3-((2-(2-(2-(2-(2-formylphenoxy)acetamido)ethoxy)ethoxy)benzyl)amino)-5-(quinolin-4-yl)benzoate (50 mg, 0.079 mmol), methyl 3-amino-5-(quinolin-4-yl)benzoate (21.96 mg, 0.079 mmol)l, and 2 mL AcOH/NaOAc buffer (pH=4.6) in methanol. Siliabond Cyanoborohydride (R66730B, Silicycle) (237 mg, 0.237 mmol) was then added and the reaction was allowed to proceed for 2 hours. The reaction was then filtered through celite, concentrated under vacuum, and then partitioned between DCM (20 mL) and saturated sodium bicarbonate (20 mL). The organic layer was collected and concentrated onto silica and purified via FLASH chromatography (10-100% EtOAc/Heptanes) to yield methyl 3-((2-(2-(2-(2-(2-(((3-(methoxycarbonyl)-5-(quinolin-4-yl)phenyl)amino)methyl)phenoxy)acetamido)ethoxy)ethoxy)-benzyl)amino)-5-(quinolin-4-yl)benzoate (40 mg, 0.045 mmol, 56.6 % yield). Once dried, methyl 3-((2-(2-(2-(2-(2-(((3-(methoxycarbonyl)-5-(quinolin-4-yl)phenyl)amino)methyl)-phenoxy)acetamido)ethoxy)ethoxy)benzyl)amino)-5-(quinolin-4-yl)benzoate (40 mg, 0.045 mmol, 56.6 % yield) was dissolved in 1 mL MeOH followed by the addition of lithium hydroxide (9.45 mg, 0.395 mmol). The reaction was stirred for 2 h and was then directly purified by HPLC to yield 2,2,2-trifluoroacetic acid compound with 3-((2-(2-(2-(2-(2-(((3-carboxy-5-(quinolin-4-yl)phenyl)amino)methyl)phenoxy)acetamido)ethoxy)ethoxy)-benzyl)amino)-5-(quinolin-4-yl)benzoic acid:2TFA as a white solid.

^1^H NMR (400 MHz, DMSO) δ 9.17 (d, *J* = 5.2 Hz, 2H), 8.23 (d, *J* = 8.5 Hz, 2H), 8.00 (ddd, *J* = 8.5, 6.8, 1.3 Hz, 2H), 7.95 – 7.92 (m, 1H), 7.90 (d, *J* = 8.6 Hz, 1H), 7.79 (d, *J* = 2.3 Hz, 1H), 7.77 (d, *J* = 2.3 Hz, 1H), 7.71 (ddt, *J* = 8.6, 7.0, 1.6 Hz, 2H), 7.47 (t, *J* = 1.9 Hz, 1H), 7.42 (t, *J* = 1.9 Hz, 1H), 7.35 – 7.30 (m, 2H), 7.28 (dd, *J* = 7.3, 1.7 Hz, 3H), 7.22 (td, *J* = 7.8, 1.7 Hz, 2H), 7.00 – 6.96 (m, 2H), 6.96 – 6.87 (m, 4H), 4.49 (s, 2H), 4.43 (s, 2H), 4.34 (s, 2H), 4.03 (dd, *J* = 5.8, 3.7 Hz, 2H), 3.64 (dd, *J* = 5.7, 3.7 Hz, 2H), 3.41 (t, *J* = 5.9 Hz, *J* = 6.0 Hz 2H), 3.20 (q, *J* = 6.0 Hz, *J* = 6.0 Hz 2H). ^13^C NMR (151 MHz, DMSO) δ 168.25, 167.95, 167.93, 158.40, 158.19, 156.56, 155.98, 150.66, 150.33, 149.59, 149.42, 148.35, 148.06, 138.48, 138.45, 132.44, 132.38, 130.13, 129.56, 128.64, 128.58, 128.48, 128.43, 128.35, 127.96, 127.74, 127.55, 127.47, 126.25, 125.95, 125.81, 125.78,121.62, 121.52, 120.97, 118.92, 117.96, 117.85, 117.20, 117.07, 115.14, 113.73, 113.70, 112.46, 112.36, 69.31, 69.12, 67.91, 67.43, 49.05, 49.06, 42.20. LC/MS: RT: 0.80, MS: 868.3 [M+1].

**Synthesis of 5,5'-((((((ethane-1,2-iylbis(oxy))bis(ethane-2,1-diyl))bis(oxy))bis(2,1-phenylene))bis(methylene))bis(azanediyl))bis(3-(quinolin-4-yl)benzoic acid (Compound 10)**

**Preparation of 2,2'-(((ethane-1,2-diylbis(oxy))bis(ethane-2,1-diyl))bis(oxy))dibenzaldehyde:** In a 40 mL scintillation vial with magnetic stirrer was added 2-hydroxybenzaldehyde (1.221 g, 10 mmol) followed by the addition of dry potassium carbonate (2.76 g, 20.00 mmol). The reaction was stirred at 80 ºC for 15 minutes to preform the potassium salt. Then (ethane-1,2-diylbis(oxy))bis(ethane-2,1-diyl) dimethanesulfonate (3.06 g, 10.00 mmol) was added and the reaction mixture was heated to 110 ºC overnight. The dimethylformamide solution was then poured onto ice (10 g) and stirred for 1 hour. The precipitate was then collected by filtration and dried under vacuum to yield 2,2'-(((ethane-1,2-diylbis(oxy))bis(ethane-2,1-diyl))bis(oxy))dibenzaldehyde (2.7 g, 7.53 mmol, 75 % yield) as a waxy solid. This material was used directly in the next step without further purification. ^1^H NMR (500 MHz, DMSO) δ 10.39 (s, 1H), 7.67 (dd, *J* = 7.7, 1.8 Hz, 1H), 7.66 – 7.60 (m, 1H), 7.23 (dd, *J* = 8.5, 0.9 Hz, 1H), 7.10 – 7.04 (m, 1H), 4.27 – 4.18 (m, 2H), 3.83 – 3.79 (m, 2H), 3.64 (s, 2H). RT: 0.92 min, MS: 359.1 [M+1].

**Preparation of 5,5'-((((((ethane-1,2-iylbis(oxy))bis(ethane-2,1-diyl))bis(oxy))bis(2,1-phenylene))bis(methylene))bis(azanediyl))bis(3-(quinolin-4-yl)benzoic acid (compound 10):** In an 8 mL vial with magnetic stirrer was added methyl 3-amino-5-(quinolin-4-yl)benzoate (78 mg, 0.279 mmol), 2,2'-(((ethane-1,2-diylbis(oxy))bis(ethane-2,1-diyl))bis(oxy))dibenzaldehyde (50 mg, 0.140 mmol), and 4mL AcOH/NaOAc buffer (pH=4.6) in methanol. Siliabond Cyanoborohydride (R66730B, Silicycle) (837 mg, 0.837 mmol) was then added (silicycle, xx) and the reaction was allowed to proceed overnight. The reaction was then filtered through celite, concentrated under vacuum, and then partitioned between DCM and saturated sodium bicarbonate. The organic layer was dried onto silica gel and purified by flash chromatography (EtOAc/heptane, 10-100%). The purified fractions were then dried and dissolved in a solution of lithium hydroxide (1797 µl, 1.797 mmol). The reaction was heated to 50 deg for 3 hours, was then dried onto celite, and was then directly purified by reverse phase HPLC (10-90% MeCN/Water with 0.1% ammonium acetate) to yield 5,5'-((((((ethane-1,2-iylbis(oxy))bis(ethane-2,1-diyl))bis(oxy))bis(2,1-phenylene))bis(methylene))bis(azanediyl))bis(3-(quinolin-4-yl)benzoic acid) (40 mg, 0.047 mmol, 33.5 % yield) (**Compound 10**) as a white solid. Also isolated in this process was xx ^1^H NMR (400 MHz, DMSO) δ 9.11 (d, *J* = 5.0 Hz, 1H), 8.20 (dd, *J* = 8.6, 1.2 Hz, 1H), 7.94 (ddd, *J* = 8.4, 6.8, 1.4 Hz, 1H), 7.86 (dd, *J* = 8.6, 1.3 Hz, 1H), 7.70 – 7.60 (m, 2H), 7.45 – 7.38 (m, 1H), 7.30 (dd, *J* = 7.8, 1.8 Hz, 1H), 7.26 (t, *J* = 1.5 Hz, 1H), 7.22 (td, *J* = 7.8, 1.8 Hz, 1H), 6.95 (d, *J* = 1.3 Hz, 1H), 6.94 – 6.89 (m, 2H), 4.33 (s, 2H), 4.02 (dd, *J* = 5.8, 3.6 Hz, 2H), 3.68 – 3.59 (m, 2H), 3.44 (s, 2H). ^13^C NMR (151 MHz, DMSO) δ 168.31, 161.41, 156.68, 156.63, 149.47, 149.25, 138.03, 136.78, 132.52, 128.47, 128.45, 127.90, 127.56, 126.38, 126.08, 124.94, 121.77, 120.97, 117.87, 116.98, 114.35, 114.12, 112.48, 69.26, 68.71, 67.97, 49.06, 46.92. LC/MS: RT: 0.84 min, MS: 855.4 [M+1].

**Synthesis of 3-((2-(2-(2-(2-(2-(((3-carboxy-5-(8-(2-(2-(2-(2',7'-difluoro-3',6'-dihydroxy-3-oxo-3H-spiro[isobenzofuran-1,9'-xanthen]-5-ylcarboxamido)ethoxy)ethoxy)acetamido)-quinolin-4-yl)phenyl)amino)methyl)phenoxy)ethoxy)ethoxy)ethoxy)benzyl)amino)-5-(quinolin-4-yl)benzoic acid, Trifluoroacetic Acid (Compound 11)**

**Preparation of 3-amino-5-(8-(2,2-dimethyl-4-oxo-3,8,11-trioxa-5-azatridecanamido)-quinolin-4-yl)benzoate:** In a 4 mL vial containing a magnetic stirrer, PdCl2dppf (45.4 mg, 0.062 mmol) and potassium acetate (243 mg, 2.481 mmol) in 1,4-Dioxane (1.6 ml) was purged with N2 for 5 min. The reaction was heated to 90°C for 1 h and was then cooled to room temperature. tert-butyl (2-(2-(2-((4-bromoquinolin-8-yl)amino)-2-oxoethoxy)ethoxy)ethyl)carbamate (290 mg, 0.620 mmol), dCl2dppf (45.4 mg, 0.062 mmol), cesium carbonate (1.550 ml, 1.550 mmol) and Water (0.400 ml) were added. The resulting mixture was purged with N2 for 5 min and was then heated to 90 °C for 2 h. The contents of the reaction were added to a 50 mL separatory funnel containing 10 mL dichloromethane and 10 mL water. The organic layer was collected and the the aqueous phase was extracted with DCM (10 mLx2). The combined organics were dried over magnesium sulfate and concentrated. The crude product was purified by FLASH chromatography (10-90% EtOAc/DCM) to afford the title compound methyl 3-amino-5-(8-(2,2-dimethyl-4-oxo-3,8,11-trioxa-5-azatridecanamido)-quinolin-4-yl)benzoate (164 mg, 0.304 mmol, 49 % yield) as an off-white solid. RT: 0.90 min, MS: 539.1 [M+1].

**Preparation of 3-((2-(2-(2-(2-(2-(((3-carboxy-5-(8-(2,2-dimethyl-4-oxo-3,8,11-trioxa-5-azatridecanamido)quinolin-4-yl)phenyl)amino)methyl)phenoxy)ethoxy)ethoxy)ethoxy)-benzyl)amino)-5-(quinolin-4-yl)benzoic acid:** In an 8 mL round bottom flask with magnetic stirrer was added methyl 3-((2-(2-(2-(2-(2-formylphenoxy)ethoxy)ethoxy)ethoxy)benzyl)amino)-5-(quinolin-4-yl)benzoate (150 mg, 0.242 mmol) and methyl 3-amino-5-(8-(2,2-dimethyl-4-oxo-3,8,11-trioxa-5-azatridecanamido)quinolin-4-yl)benzoate (130 mg, 0.242 mmol) followed by the addition of 4 mL 1M acetic acid/sodium acetate buffered at (pH 4.6) in MeOH. Then Siliabond Cyanoborohydride (R66730B, Silicycle) (967 µl, 0.967 mmol) was added in one portion and the reaction was allowed to proceed at room temperature overnight. The contents were then filtered through celite and concentrated into a 50 mL round bottom flask. To the crude solid was added acetone (15 mL) and the mixture was vigorously stirred for one hour. The acetone solution was filtered and dried to yield a sticky solid that was then dissolved in 2 mL 1N LiOH in MeOH and stirred at 50 deg for 3h. The reaction mixture was purified by reverse phase HPLC (0.1% Ammonium Acetate) to yield 3-((2-(2-(2-(2-(2-(((3-carboxy-5-(8-(2,2-dimethyl-4-oxo-3,8,11-trioxa-5-azatridecanamido)quinolin-4-yl)phenyl)amino)methyl)phenoxy)ethoxy)ethoxy)ethoxy)benzyl)amino)-5-(quinolin-4-yl)benzoic acid (65 mg, 0.058 mmol, 24.12 % yield) as a white solid.

^1^H NMR (501 MHz, DMSO) δ 12.9 (br s, 2H) 10.75 (d, *J* = 7.1 Hz, 1H), 9.05 (d, *J* = 4.9 Hz, 1H), 8.90 (dd, *J* = 4.5, 1.5 Hz, 1H), 8.67 (dd, *J* = 7.6, 1.4 Hz, 1H), 8.14 (d, *J* = 8.2 Hz, 1H), 7.89 (ddd, *J* = 8.3, 6.8, 1.3 Hz, 1H), 7.83 – 7.80 (m, 1H), 7.62 – 7.59 (m, 1H), 7.49 – 7.47 (m, 1H), 7.46 (d, *J* = 7.8 Hz, 1H), 7.41 (dd, *J* = 8.6, 1.4 Hz, 1H), 7.38 (q, *J* = 3.3, 2.6 Hz, 1H), 7.34 (t, *J* = 1.9 Hz, 1H), 7.28 (d, *J* = 1.8 Hz, 1H), 7.27 (d, *J* = 1.8 Hz, 1H), 7.24 – 7.16 (m, 4H), 6.96 – 6.91 (m, 4H), 6.87 (dt, *J* = 10.8, 2.0 Hz, 2H), 6.74 (s, 1H), 4.30 (s, 2H), 4.29 (s, 2H), 4.23 (s, 2H), 3.99 (dd, *J* = 5.0 Hz, *J* = 10.4 Hz 4H), 3.78 (m, 4H), 3.70 – 3.67 (m, 4H), 3.60 (m, 4H), 3.48 (t, *J* = 6.1 Hz, 2H), 3.41 (s, 4H), 3.10 (dd, *J* = 6.1 Hz, *J* = 11.4 Hz 2H), 1.32 (s, 9H).

Preparation of **3-((2-(2-(2-(2-(2-(((3-carboxy-5-(8-(2-(2-(2-(2',7'-difluoro-3',6'-dihydroxy-3-oxo-3H-spiro[isobenzofuran-1,9'-xanthen]-5-ylcarboxamido)ethoxy)ethoxy)acetamido)-quinolin-4-yl)phenyl)amino)methyl)phenoxy)ethoxy)ethoxy)ethoxy)benzyl)amino)-5-(quinolin-4-yl)benzoic acid, Trifluoroacetic Acid (Compound 11):**

Note: Attempts at mild amine deprotection with prolonged reaction times resulted in oxidative deamination and amine salts were not stable for prolonged periods of time on the bench.

In an amber 4 mL vial was added 3-((2-(2-(2-(2-(2-(((3-carboxy-5-(8-(2,2-dimethyl-4-oxo-3,8,11-trioxa-5-azatridecanamido)quinolin-4-yl)phenyl)amino)methyl)phenoxy)ethoxy)-ethoxy)ethoxy)benzyl)amino)-5-(quinolin-4-yl)benzoic acid (25 mg, 0.022 mmol) and trifluoroacetic acid (173 µl, 2.242 mmol) the reaction was stirred for 1 minute and then concentrated to dryness by rotovap to yield 3-(8-(2-(2-(2-aminoethoxy)ethoxy)acetamido)-quinolin-4-yl)-5-((2-(2-(2-(2-(2-(((3-carboxy-5-(quinolin-4-yl)phenyl)amino)methyl)phenoxy)-ethoxy)ethoxy)ethoxy)benzyl)amino)benzoic acid, 2 Trifluoroacetic Acid (17 mg, 0.014 mmol) with approximately ~90% purity. Once dried 2 mL toluene was added and compound was concentrated again to remove excess trifluoroacetic acid. To this residue was added

2,5-dioxopyrrolidin-1-yl 2',7'-difluoro-3',6'-dihydroxy-3-oxo-3H-spiro[isobenzofuran-1,9'-xanthene]-5-carboxylate (5 mg, 9.82 µmol) and 1 mL of an anhydrous DIEA (2%) in DMSO solution. The contents were shaken at room temperature for 24 h at which time 2 mL 90% DMSO/water was added and the reaction was purified by reverse phase HPLC utilizing a Waters Deltapak C18 200 x 25 mm column eluted with a gradient of 5% A (0.1% TFA-water):B (MeCN) to 95% A:B to yield 3-((2-(2-(2-(2-(2-(((3-carboxy-5-(8-(2-(2-(2-(2',7'-difluoro-3',6'-dihydroxy-3-oxo-3H-spiro[isobenzofuran-1,9'-xanthen]-5-ylcarboxamido)ethoxy)ethoxy)-acetamido)-quinolin-4-yl)phenyl)amino)methyl)phenoxy)ethoxy)ethoxy)ethoxy)benzyl)amino)-5-(quinolin-4-yl)benzoic acid, Trifluoroacetic Acid (**Compound 11**) (13.1 mg, 8.8 µmol, 63% yield) as a bright yellow solid. RT: 0.90 min, MS: 1409.2 [M+1].

**Characterization of (E)-7-(prop-1-en-1-yl)-1H-indazol-3-amine (Compound 4)**

^1^H NMR (500 MHz, DMSO) δ 11.48 (s, 1H), 7.54 (dd, *J* = 8.0, 0.9 Hz, 1H), 7.26 (d, *J* = 7.1 Hz, 1H), 6.87 (t, *J* = 7.5 Hz, 1H), 6.75 – 6.58 (m, 1H), 6.39 (dq, *J* = 15.8, 6.5 Hz, 1H), 5.32 (s, 2H), 1.91 (dd, *J* = 6.6, 1.7 Hz, 3H).

**Characterization of 5-(5-(benzylamino)pyridin-3-yl)-N-(2-morpholinoethyl)-1H-indazol-3-amine (Compound 5)**

^1^H NMR (600 MHz, DMSO) δ 11.49 (s, 1H), 8.05 (d, *J* = 2.0 Hz, 1H), 7.98 (d, *J* = 1.7 Hz, 1H), 7.92 (d, *J* = 2.7 Hz, 1H), 7.46 (dd, *J* = 8.6, 1.7 Hz, 1H), 7.43 – 7.39 (m, 2H), 7.34 (dd, *J* = 8.5, 6.9 Hz, 2H), 7.30 (d, *J* = 8.6 Hz, 1H), 7.27 – 7.21 (m, 1H), 7.13 (t, *J* = 2.3 Hz, 1H), 6.55 (t, *J* = 6.1 Hz, 1H), 5.91 (t, *J* = 5.8 Hz, 1H), 4.39 (d, *J* = 6.0 Hz, 2H), 3.60 (t, *J* = 4.6 Hz, 4H), 3.40 (q, *J* = 6.4 Hz, 2H), 2.60 (t, *J* = 6.9 Hz, 2H), 2.45 (s, 4H). ^13^C NMR (151 MHz, DMSO) δ 150.82, 145.11, 141.72, 140.10, 136.97, 135.71, 134.06, 128.86 (2C), 127.87, 127.80 (2C), 127.30, 126.03, 118.69, 116.01, 114.80, 110.35, 66.66 (2C), 59.01, 54.01 (2C), 46.57, 40.82.

**Characterization of *N*-benzyl-6-chloro-5-(quinolin-5-yl)pyridin-3-amine (Compound 6)**

^1^H NMR (400 MHz, DMSO) δ 8.93 (dd, *J* = 4.1, 1.7 Hz, 1H), 8.09 (dt, *J* = 8.5, 1.1 Hz, 1H), 7.92 (d, *J* = 3.0 Hz, 1H), 7.83 (dd, *J* = 8.5, 7.1 Hz, 1H), 7.75 (ddd, *J* = 8.5, 1.7, 0.9 Hz, 1H), 7.54 – 7.44 (m, 2H), 7.41 – 7.31 (m, 4H), 7.30 – 7.23 (m, 1H), 7.03 (d, *J* = 3.0 Hz, 1H), 6.84 (t, *J* = 6.0 Hz, 1H), 4.34 (d, *J* = 5.6 Hz, 2H). ^13^C NMR (101 MHz, DMSO) δ 151.07, 148.01, 144.77, 139.39, 136.45, 135.92, 134.55, 133.95, 133.50, 129.87, 129.48, 128.91 (2C), 128.05, 127.87 (2C), 127.44, 126.39, 123.71, 122.37, 46.56.

**Characterization of 2-hydroxy-5-(6-methylquinolin-5-yl)benzoic acid (Compound 7)**

^1^H NMR (400 MHz, DMSO) δ 9.02 (dd, *J* = 4.6, 1.6 Hz, 1H), 8.09 (dd, *J* = 8.7, 0.9 Hz, 1H), 8.05 (dt, *J* = 8.6, 1.2 Hz, 1H), 7.89 (d, *J* = 8.8 Hz, 1H), 7.69 – 7.62 (m, 2H), 7.47 (dd, *J* = 8.4, 2.3 Hz, 1H), 7.17 (d, *J* = 8.5 Hz, 1H), 2.29 (s, 3H). ^13^C NMR (101 MHz, DMSO) δ 172.08, 160.91, 147.79, 143.15, 137.99, 137.61, 137.51, 136.06, 134.56, 131.89, 128.21, 128.01, 125.56, 122.29, 118.23, 113.82, 20.81.
